# Decoding type 5 diabetes using spatial omics: microarchitectural and molecular mechanisms of malnutrition-associated diabetes

**DOI:** 10.3389/fendo.2026.1822644

**Published:** 2026-06-05

**Authors:** Anusha Komati, Kiranmai Mandava, Ajay Anand

**Affiliations:** 1 Pharmacology Department, St. Pauls College of Pharmacy, Turkayamjal, Telanagana, India; 2 Department of Pharmaceutical Chemistry, St. Pauls College of Pharmacy, Turkayamjal, Telanagana, India; 3 Department of Internal Medicine, University of Iowa, Iowa City, IA, United States

**Keywords:** malnutrition-associated diabetes, type 5 diabetes mellitus, spatial omics, molecular mechanisms, gut-microbiome

## Abstract

This review advocates for the formal recognition of Type 5 Diabetes Mellitus, a distinct, neglected form of non-autoimmune, lean diabetes predominantly affecting low-income and middle-income populations with a history of early-life malnutrition. Characterized by impaired insulin secretion amid preserved insulin sensitivity, this phenotype exhibits unique microarchitectural alterations within pancreatic islets and is strongly linked to malnutrition-induced epigenetic reprogramming and gut microbiota dysbiosis. Recent advancements in spatial omics technologies have illuminated region-specific gene expression, inflammatory pathways, and cellular remodeling in pancreatic tissue, offering novel insights into its pathophysiology. Despite its global prevalence, especially in resource-constrained settings, Type 5 diabetes remains underdiagnosed and poorly understood, hindering the development of tailored diagnostic criteria and therapeutic strategies. This review synthesizes current epidemiological, mechanistic, and molecular evidence, emphasizing the imperative for establishing standardized classification, research collaborations—including the formation of the IDF Type 5 Diabetes Working Group—and region-specific management protocols. Recognizing this disease entity is critical for advancing health equity, improving clinical outcomes, and guiding future research in metabolic disease frameworks.

## Graphical Abstract

**Figure:**
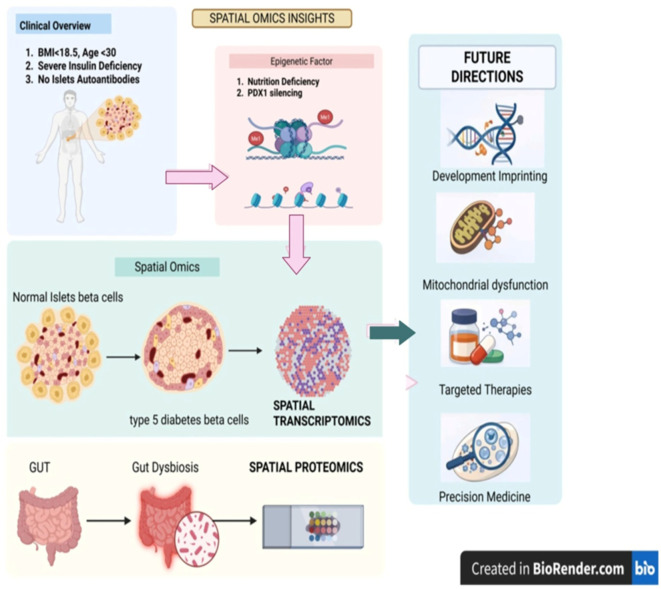

## 1 Introduction

Diabetes remains a growing public health challenge globally, with a significant rise in the number of individuals affected anticipated in low-income and middle-income countries (LMICs), which are contending with both undernutrition and overnutrition. The escalating challenges of obesity and aging populations are significant risk factors for the rising incidence of type 2 diabetes; however, undernutrition also seems to play a role, as atypical forms of non-type-1 diabetes have been documented in young, lean individuals (BMI <18.5 kg/m², age <30 years) with a history of undernutrition (1). This variant of diabetes was initially recorded in The Lancet in 1955 by P Hugh-Jones, who designated it as J-type diabetes, based on his observations of young, underweight individuals in Jamaica exhibiting insulin-resistant diabetes without a tendency towards ketoacidosis (2). Initial clinical observations indicated that individuals with J-type diabetes seemed to necessitate substantial insulin doses; however, more comprehensive physiological studies have revealed that the majority of these individuals possess normal insulin sensitivity, coupled with significant impairments in insulin secretion (3). In 1985, the WHO identified J-type diabetes as a separate category, termed malnutrition-related diabetes, but retracted its official categorization in 1999 due to inadequate evidence linking it to malnutrition. Five Reports from numerous low- and middle-income countries (LMICs), such as India, Pakistan, Bangladesh, Uganda, Ethiopia, Rwanda, Nigeria, and Indonesia, substantiate the presence of an atypical diabetes phenotype in lean individuals, correlating with low socioeconomic status and a history of prolonged undernutrition. Furthermore, the tenth and eleventh iterations of the ICD, namely ICD-10 (code: E12.9) and ICD-11 (code: E12.11), also maintain classification codes for diabetes associated with malnutrition.

According to research, including those from the Diabetes Atlas, it is projected that 25 million individuals in LMICs have diabetes (4, 5). A 2024 study of two decades of trends in lean individuals with diabetes indicates a prevalence increase comparable to that of type 2 diabetes among South Korean adults (6). Nevertheless, owing to disputes regarding nomenclature, insufficient acknowledgment by the WHO, and its prevalence in economically disadvantaged regions of LMICs, this atypical diabetes variant has garnered significantly less attention compared to type 1 and type 2 diabetes, resulting in inadequate phenotyping and ambiguous classification (7). The lack of prospective cohort studies and mechanistic research has resulted in insufficient comprehension of the natural history and pathophysiology of this condition. Moreover, the lack of adequate data for this type of diabetes complicates the establishment of diagnostic criteria and risk factors relevant to various groups. This condition may be misdiagnosed due to diagnostic overlap with other atypical diabetes variants (e.g., fibrocalculous pancreatic diabetes and antibody-negative type 1 diabetes) (**Table 1**), particularly in resource-constrained environments, resulting in under-reporting and persistent diagnostic oversight in global research and policy initiatives. The efficacy of insulin and oral hypoglycemic medications in persons with this type of diabetes has not been assessed in intervention trials, presenting management difficulties for healthcare providers. Consequently, misdiagnosis and underdiagnosis are likely to have adversely affected the clinical care and lives of millions globally. Misdiagnosing these young, slender persons with type 1 diabetes may result in significant iatrogenic hypoglycemia.

**Table 1:** Differences between type 1, 2 and type 5 diabetes.

Parameter	Type 1 diabetes mellitus (T1DM)	Type 2 diabetes mellitus (T2DM)	Type 5 diabetes mellitus (T5DM)
Primary Etiology	Autoimmune destruction of pancreatic β-cells	Insulin resistance with relative insulin deficiency	Early-life malnutrition–induced β-cell dysfunction
Pathophysiology	Absolute insulin deficiency	Insulin resistance + progressive β-cell failure	Impaired insulin secretion with preserved insulin sensitivity
Autoantibodies (GAD, IA-2)	Present	Absent	Absent
Age at Onset	Childhood or adolescence	Middle to late adulthood	Young adulthood (usually <30 years)
Body Mass Index (BMI)	Normal or low	Overweight or obese	Low BMI (<18.5 kg/m²)
Association with Malnutrition	No	No	Strong (early-life and chronic undernutrition)
Insulin Sensitivity	Normal or increased	Reduced (insulin resistance)	Preserved or normal
C-Peptide Levels	Very low or absent	Normal or high initially, declines later	Low but detectable
Ketoacidosis Risk	High	Low (except stress states)	Rare
Pancreatic Imaging	Normal	Normal or fatty infiltration	Normal (no calcification or ductal changes)
Gut Microbiota Involvement	Limited evidence	Strong association	Strong association (malnutrition-driven dysbiosis)
Epigenetic Alterations	Minimal evidence	Present (obesity-related)	Prominent (DNA methylation, histone modifications, miRNAs)
Socioeconomic Association	None specific	Urbanization, sedentary lifestyle	Low socioeconomic status, LMICs
Geographic Distribution	Global	Global	Predominantly LMICs
Primary Treatment Strategy	Lifelong insulin therapy	Lifestyle + oral agents ± insulin	Nutritional rehabilitation ± low-dose insulin/oral agents
Risk of Hypoglycemia	High	Moderate	High if misclassified as T1DM
WHO/IDF Recognition	Fully recognized	Fully recognized	Officially recognized by IDF (2025)
Representative References	9, 17, 80	4, 5, 78	1, 3, 7, 69, 73

Table demonstrates differences in clinical, metabolic and pathophysiology features of Type 1, Type 2 and Type 5 Diabetes Mellitus. Type 1 diabetes (T1DM): autoimmune destruction of pancreatic beta cells leading to absolute insulin deficiency. Type 2 diabetes (T2DM): insulin resistance with impaired or relative insulin deficiency, often associated with obesity and metabolic syndrome. Type 5 diabetes (malnutrition-related diabetes mellitus): chronic undernutrition (low BMI), impairment of beta-cell function without autoimmunity, marked by reduced insulin sensitivity (reduced C-peptide). Differences in clinical features, metabolic characteristics, pathophysiology, clinical presentation, complications and management strategies for Type 1, Type 2 and Type 5 Diabetes Mellitus.

On January 8 and 9, 2025, the 39 signatories of the consensus declaration convened in Vellore, India, to present and evaluate studies related to this overlooked kind of diabetes affecting lean and undernourished adults. The participants comprised researchers who had thoroughly investigated lean, undernourished diabetic patients in low- and middle-income countries, as well as leaders from worldwide diabetes organizations and internationally esteemed experts in several fields of diabetes research. Each participant presented pertinent research findings, engaged in structured discussions to critically evaluate the existing literature, deliberated a differential diagnosis to exclude other recognized forms of diabetes with a low BMI, identified research gaps, and explored opportunities for future research concerning the recognition of a distinct classification and the understanding of the pathophysiology of this type of diabetes. Subsequent to these conversations, the signatories unanimously concurred that a different, non-autoimmune variant of diabetes in undernourished individuals necessitated a new classification for diagnostic and therapeutic purposes (the “Vellore Declaration”). This Viewpoint outlines the evaluated data that resulted in international consensus, enumerates the disease’s shared characteristics, and suggests pathophysiological mechanisms, management protocols, and research goals for the future. Acknowledging that alternative classifications have been identified as type 3 diabetes and type 4 diabetes in existing literature, type 5 diabetes has been suggested as the designation for this kind of diabetes. The International Diabetes Federation (IDF) officially confirmed this name at its World Diabetes Congress in April 2025. During this conference, IDF President Peter Schwarz formally inaugurated the IDF’s Type 5 Diabetes Working Group, assigned with the responsibility of formulating precise diagnosis criteria and treatment protocols for this ailment.

In light of the growing number of studies of the transcriptome, proteome and epigenome, while always preserving the structural integrity of the biological samples, the spatial omics methods give us completely new knowledge on the regulation of the biological processes occurring in the pancreas. The spatial transcriptomics, as well as highly multiplexed imaging, add new knowledge about the immune cells infiltrating the pancreas and on the functions of the endocrine cells, by characterizing the microvascular niche of the islets, *in situ*, in the intact tissue. literature has shown that even within the same islet, pancreatic β-cells have very different functions at different locations along different regions (i.e. different combinations of metabolic pathways) and within stress responses networks and genes.

Although transcriptomics has progressed, the understanding of the pathophysiology of malnutrition-associated diabetes (Type 5 diabetes mellitus, T5DM) at the level of tissue structure and spatially regulated β-cell dysfunction remains limited. Prior research utilizing bulk and single-cell RNA sequencing has identified β-cell diversity and metabolic changes in diabetes (8–11); however, these methods lack spatial resolution and thus fail to elucidate how nutrient deprivation, environmental signals, and interactions between cells within pancreatic tissue lead to β-cell failure. This constitutes a significant gap in knowledge, especially in T5DM, where it is proposed that chronic undernutrition affects β-cell mass and function through nutrient-sensitive signaling pathways like mTOR and crucial transcriptional regulators such as PDX1 (12), yet the spatial context of these changes is still largely unexamined.

This study aims to fill the existing gap by utilizing spatial omics to explore the impact of tissue microarchitecture on β-cell dysfunction in malnutrition scenarios. Unlike previous studies that focused on descriptive mapping, this research combines spatial gene expression with quantitative spatial analysis and interaction modeling to pinpoint spatially resolved patterns of β-cell dysfunction, nutrient-sensitive signaling deficiencies, and unique cellular niches linked to T5DM pathology. Specifically, the study sheds light on the mechanisms by which limited nutrient availability may spatially inhibit mTORC1 signaling and PDX1 activity in certain islet areas, resulting in reduced insulin production and β-cell survival. Additionally, the discovery of spatially organized interactions among β-cells, immune cells, and stromal components underscores the significance of the microenvironment in influencing disease progression, a factor that non-spatial methods cannot capture.

This research crucially reveals that β-cell dysfunction in T5DM is not evenly distributed but is influenced by specific metabolic and signaling gradients within the pancreas, thereby connecting malnutrition to spatially distinct cellular dysfunction. By advancing beyond descriptive atlases to identify mechanistic, spatially detailed pathways that contribute to β-cell impairment, this study introduces a new framework for comprehending T5DM pathogenesis. These insights not only address a significant gap in current diabetes research but also lay the groundwork for creating targeted therapeutic strategies that focus on the microenvironment in malnutrition-related diabetes.

## 2 Common features

**Figure 1:**
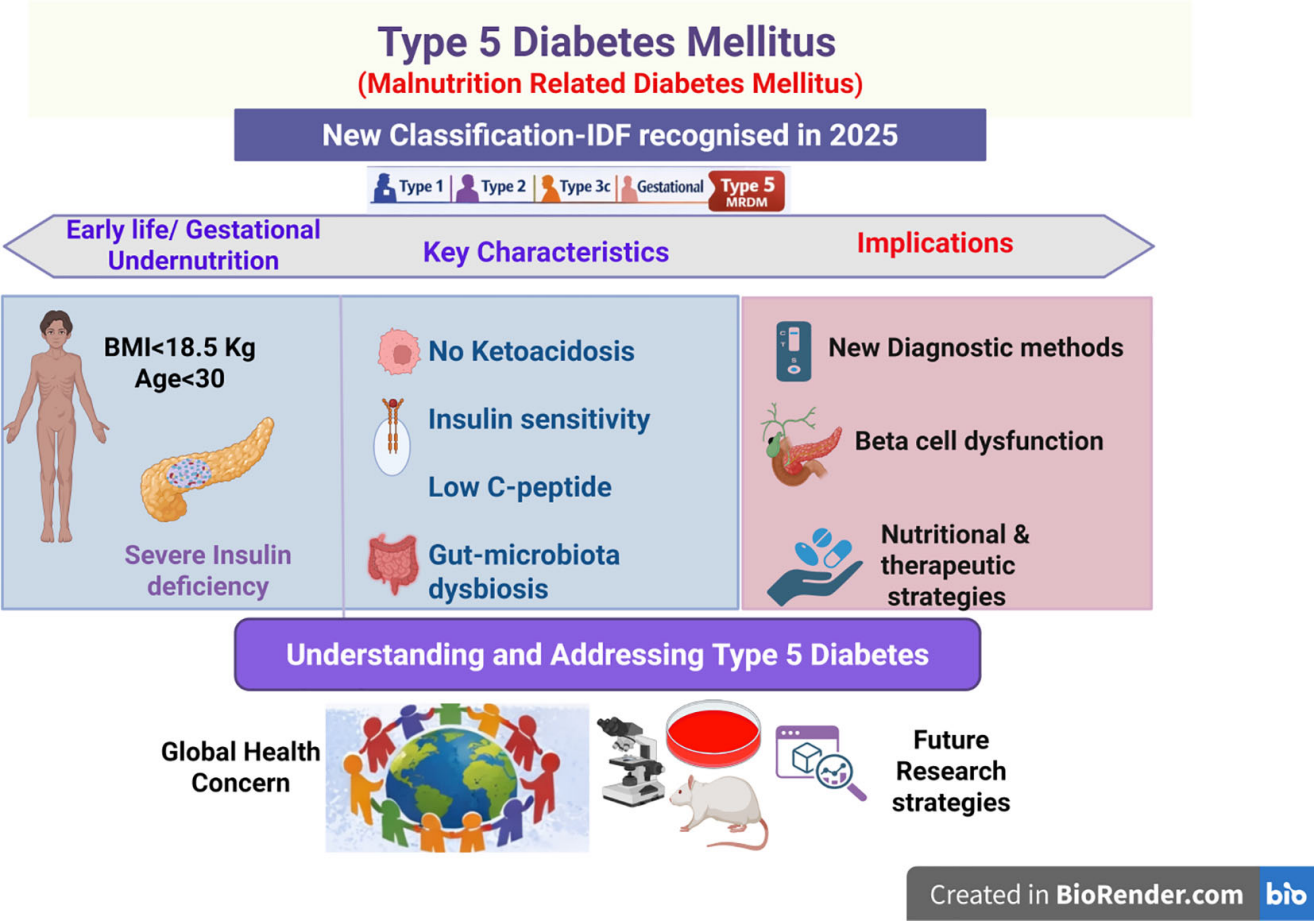
Overview of type 5 diabetes. Illustrates new emerging Type 5 Diabetes Mellitus classified by the International Diabetes Federation in 2025. These subjects are characterized by early-life or gestational undernutrition. They are generally lean (BMI <18.5 kg/m²), young (<30 years), severely deficient in insulin, non-ketosis, and highly insulin sensitive with low C-peptide levels and disturbances of gut microbiota (indicated by). This figure also highlights several important clinical points including the need for better diagnostic tests for Type 5 Diabetes, recognition of β-cell dysfunction, potential nutritional and therapeutic approaches to Type 5 Diabetes and their distinctiveness from Type 1 and Type 2 Diabetes. In addition, it underlines global relevance and future research directions of Type 5 Diabetes including experimental models and further studies to clarify mechanisms which might cause complications in Type 5 Diabetes.

In the last seventy years, the phenotype referred to as type 5 diabetes has gained global recognition, predominantly among underweight individuals in resource-limited environments. Extensive physiological researches in lean individuals with diabetes, following the meticulous exclusion of other recognized kinds of diabetes, have investigated the metabolic properties of these individuals utilizing advanced technologies (1–3). These investigations, with others employing other experimental methodologies, have cumulatively recorded patients exhibiting compromised insulin production, diminished C-peptide levels, and normal insulin sensitivity, distinguishing them from individuals with type 2 diabetes (13–21) (**Figure 1**). Nevertheless, insulin levels in these patients are not as diminished as those seen in individuals with type 1 diabetes, and ketoacidosis is typically not documented (17, 22). Research indicates that these people frequently possess a history of early-life undernutrition, which persists into maturity, with the disease typically identified in the third decade of life. Individuals with a lean physique who had positive autoantibodies characteristic of type 1 diabetes, including antibodies against GAD-65 and IA-2, have been removed from our classification. Pancreatic imaging in patients with type 5 diabetes should reveal no signs of pancreatic calcifications, ductal hypoplasia, or ductal dilatation, as these characteristics are indicative of fibrocalculous pancreatic diabetes. Utilizing dual-energy absorptiometry or bioimpedance measurement, these individuals exhibit reduced total and truncal fat mass, especially hepatic lipids (1, 3). Moreover, some sociodemographic characteristics define type 5 diabetes. Research indicates that the majority of patients with type 5 diabetes are from low socioeconomic backgrounds and live in rural regions, characterized by insufficient dietary consumption of proteins and calories. The characteristics frequently and sporadically linked to type 5 diabetes are summarized in the panel.

While not all the previously mentioned characteristics are exhibited by every individual with type 5 diabetes, their presence would facilitate the differentiation of this diabetes subtype from others, including type 1 diabetes; type 2 diabetes associated with weight loss due to uncontrolled hyperglycemia and inconsistent medication; diabetes resulting from pancreatic disorders, such as fibrocalculous pancreatic diabetes; maturity-onset diabetes of the young; hereditary lipodystrophic disorders; and ketosis-prone diabetes (19, 23).

T1DM is clinically identified by its early onset and reliance on insulin, metabolically by a complete lack of insulin, and pathologically by the autoimmune destruction of β-cells, with detectable autoantibodies such as GAD and IA-2 (24, 25). In contrast, T2DM is associated with being overweight or obese, insulin resistance, and a gradual decline in β-cell function, with pathological characteristics including islet amyloid deposits and persistent low-grade inflammation (26). LADA is an intermediate form that begins in adulthood, features autoantibodies, and slowly progresses to insulin dependence, indicating a slower autoimmune β-cell loss compared to T1DM (27). MODY is a monogenic type of diabetes, usually appearing at a young age, characterized by maintained β-cell function, no autoimmunity, and specific genetic mutations that impact insulin secretion (28).

Conversely, T5DM is identified by a unique set of criteria based on chronic undernutrition. Clinically, individuals with this condition are generally thin, having experienced malnutrition or a low body mass index, often due to nutritional deprivation in early life. Metabolically, T5DM is marked by significant impairment in insulin secretion while maintaining relatively normal insulin sensitivity, setting it apart from the insulin resistance observed in T2DM. Pathologically, there is a decrease in β-cell mass, pancreatic atrophy, and hindered β-cell proliferation, frequently associated with nutrient-sensitive signaling issues such as mTOR pathway dysregulation and epigenetic changes in crucial genes like PDX1. Notably, the absence of autoimmune markers distinguishes T5DM from T1DM and LADA, while the lack of genetic mutations sets it apart from MODY. Defining these criteria based on recent studies is crucial to prevent misclassification and to acknowledge T5DM as a distinct pathophysiological category within the diabetes spectrum.

## 3 Malnutrition induced metabolic reprogramming

Malnutrition is a significant issue that impacts the functioning of the immune system in children. Malnutrition encompasses a broad spectrum of diseases, including wasting, stunting, underweight, obesity, the dual burden of stunting and overweight, and severe protein-energy malnutrition (Kwashiorkor or Marasmus). Malnutrition is associated with various factors, including socioeconomic position, age, education level, gender, family size, and geographical location (29). The dominant agreement, however, is that poverty is the primary cause of malnutrition (30). In low-income countries, the incidence of these nutritional deficits can reach 70–80% among children aged 6 months to 5 years (31). A nutritional assessment in Southeast Asia offered an overview of the micronutrient status in about 13,000 children aged 6 months to 12 years (32–38).

The World Health Organization (WHO) defines malnutrition as deficiencies, excesses, or imbalances in an individual’s energy and nutritional consumption. It is categorized into three primary types: undernutrition (comprising wasting, stunting, and underweight), micronutrient-related malnutrition (entailing deficiencies or surpluses of vital vitamins and minerals), and conditions such as overweight, obesity, and diet-related noncommunicable diseases, including cardiovascular disease, ischemic stroke, specific cancers, and diabetes. They highlighted that 60 million adults and 150 million children suffer from malnutrition, while over two billion individuals are categorized as overweight or obese.

Micronutrient malnutrition or “hidden hunger” keeps children and pregnant women in developing countries in a perpetual state of inadequate nutrition, where their bodies lack required nutrients to perform basic functions necessary for healthy development. The world’s most significant micronutrient deficiencies include iodine, vitamin A and iron deficiencies, which affect approximately 2 billion people worldwide. These deficiencies arise from a number of factors including food inadequacy and the effects of climate change on agriculture, which are addressed through food fortification, vitamin and mineral supplementation and increasing dietary diversity to help alleviate the global issues of micronutrient malnutrition. • Iodine deficiency is the most common dietary deficiency leading to primary mental deficiency. Vitamin A and Vitamin D are also important for immune tolerance, gut integrity and pancreatic β-cell function. Iron and Zinc are required for new insulin synthesis as well as for other antioxidant defenses and innate immune functions. Vitamin B12 and Folate are required for mitochondrial biogenesis and for the epigenetic regulation of glucose metabolism. All of these are also characteristics of MRDM (39–42).

### 3.1 Gut-malnutrition axis

**Figure 2:**
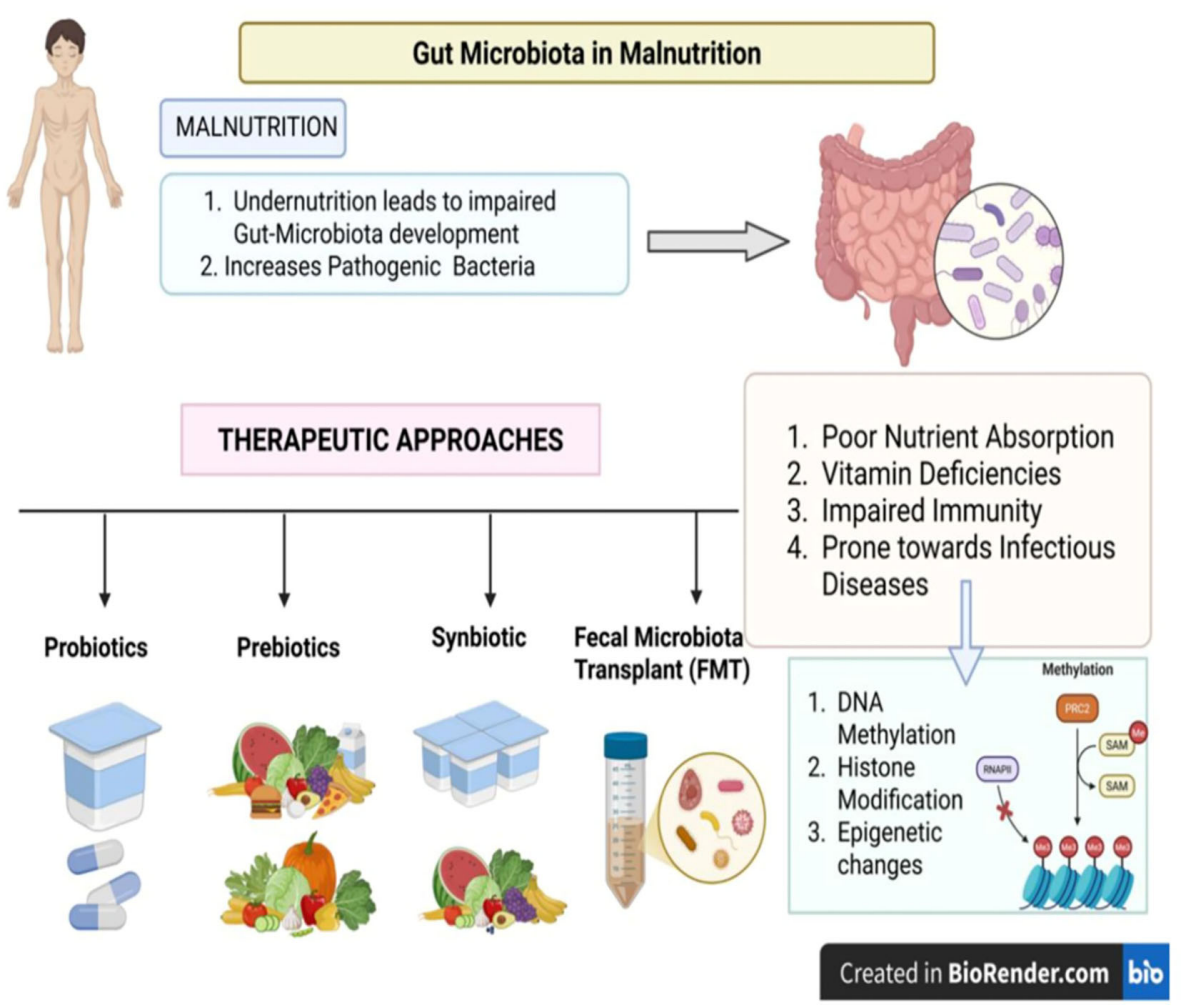
Gut microbiota in malnutrition. Displays malnutrition induced gut dysbiosis and associated complications and mechanistic insights. It also depicts treatment approaches. This figure illustrates how malnutrition affects the normal development of the gut microbiota, reducing diversity and increasing the proportion of potentially pathogenic organisms resulting in gut dysbiosis. It shows how the impaired intestinal function results in reduced nutrient absorption, inadequate levels of essential vitamins and minerals, impaired immune function and increased risk of infection. The impact of malnutrition on gut microbiota is mediated by epigenetic mechanisms such as DNA methylation and histone modification. The diagram details treatments used to restore the balance of the gut microbiota, improving gut health and increasing nutrient utilization for better health outcomes. Treatments include probiotics, prebiotics, synbiotics and fecal microbiota transplantation (FMT).

Undernutrition, particularly during early childhood, critically disrupts the establishment, maturation, and functional capacity of the gut microbiota, producing long-lasting consequences for host metabolism and immune competence. A poor calorie and nutrient supply, resulting in stunted growth, delayed brain development and immunosuppression, with the children being more prone to recurrent and severe infections. Recently it was also shown that undernourished children carry a low diversity atypical gut microbiota with an overabundance of pathogens thereby perpetuating a cycle of malabsorption and immunodeficiency (43) (**Figure 2**).

Adult undernutrition is a common problem in the elderly and in those suffering from chronic illness due to decreased appetite, inadequate nutrition to meet increased energy needs associated with the illness or infection and prolonged hospitalization (44). Alterations in the gut microbiota, involving changes to either the microbial community or its metabolic activity, and a decrease in beneficial commensal bacteria have been associated with a wide variety of diseases including inflammatory bowel disease, cardiovascular disease, diabetes, cancer and neuropsychiatric disorders (45–49). The relationship between malnutrition and microbiota imbalance is bidirectional as malnutrition and microbiota imbalance tend to exacerbate each other (50).

The gut microbiota is involved in the breakdown of the macronutrients in the diet, vitamin synthesis, short-chain fatty acids (SCFAs) production, and the maintenance of the integrity of the intestinal barrier. Malnutrition-associated alterations in microbial composition impair these processes, leading to ineffective nutrient extraction and metabolic inefficiency even when dietary intake is partially restored. Dysbiosis is frequently accompanied by chronic low-grade intestinal inflammation, anorexia, muscle catabolism, and disease-associated malnutrition (51). Overgrowth of pathogens leads to damage of the intestinal epithelium, increased permeability and severe diarrhea, which in turn prevents effective nutrient absorption.

Protein-energy malnutrition (PEM) is known to have many effects on the microbial-host interaction in the gastrointestinal (GI) tract. In this study it was discovered that a low protein diet can lead to low levels of tryptophan, resulting in low niacin levels. Niacin or vitamin B3 is required to switch on the genes of the antimicrobial peptides in the ileal epithelium, thus a niacin deficiency leads to an impaired mucosal barrier, which increases the susceptibility of the host for colonization by pathogens and contributes to dysbiosis.

Gut microbiota development starts at birth and is determined by several factors including the mode of delivery, breastfeeding and diet. The gut microbiota of healthy infants undergoes a dynamic process of maturation during the first years of life, resulting in an adult like microbial community in terms of both structure and function. Most studies indicate that this maturation process is completed between 2 and 3 years of age (52). The maturation of gut microbiota in a malnourished child is also altered. For example, in gut microbiota from children suffering from severe nutritional deficits the microbiota was reported to be immature, and the numbers of bacteria such as *Bifidobacterium* and *Bacteroides* were depressed, essential for the proper assimilation of carbohydrates, proteins, and thus the production of short chain fatty acids (SCFAs) (53). This arrested microbial maturation limits energy harvest and nutrient utilization, thereby reinforcing the malnourished state.

Protein and amino acid deficiency may affect B cell and plasma cell function. The mTOR signaling network is sensitive to amino acid availability and a low protein diet can therefore restrict amino acid availability to meet the demands required for B and plasma cell function and for the production of mucosal antibody secretory immunoglobulin A (sIgA) (54–57). However, little direct evidence exists to confirm a link between decreased mTOR activity in the mucosa and decreased sIgA. Toll-like receptors (TLRs) are key receptors for the recognition of pathogen-associated molecular patterns (PAMPs) and the initiation of an immune response. Low protein and amino acid levels can reduce expression of these receptors at the mucosal surface and decrease the MyD88/NF-κB mediated cytokine response and thus mucosal immunity. This decrease in TLR-mediated immunity can result in alterations to the gut microbiota (dysbiosis) (58, 59). sIgA is an immunoglobulin (Ig) isotype localized to the mucosal surface that acts as a mucosal coat to prevent microbial colonization of the mucosa. Malnutrition is therefore known to decrease sIgA production (60). The nuclear factor kappa-light-chain-enhancer of activated B cells (NF-κB) is a transcription factor crucial for the immune response and for IgA class switching in B cells (61). Other nutrients, including vitamins A and D, have also been shown to be necessary for NF-κB activation (62). Furthermore, the vitamin A derivative retinoic acid is required for IgA production. Decreased regulation of the NF-κB pathway in states of malnutrition is therefore associated with decreased sIgA production and a decreased gut mucosal barrier function leading to a decrease in microbial diversity (63).

### 3.2 Microbiological memory

Microbiological memory (MM) is the late persistence of epigenetic and transcriptional effects elicited by previous microbial and nutritional stimuli. Therefore MM could relate the non-genetic hereditary character of the metabolic effects of early life environmental factors. We showed that the gut microbiota is dynamic and versatile in response to diverse stimuli including dietary components, antibiotics and malnutrition, especially during early-life, when the epigenetic regulation is at its maximum plasticity. Using germ-free and antibiotic-treated models, we showed that a simple dysbiotic condition, which can be induced by changing the gut microbiota composition by various means, can by itself promote increased fat mass accumulation, metabolic alterations and decrease microbial diversity, all effects being long-lasting even after their inducing alterations have been removed.

Maternal undernutrition affects epigenetic gene regulation through DNA methylation, histone modification and microRNA (miRNA) involving genes involved in beta-cell development and function as well as glucose metabolism and resulting in pancreatic beta-cell dysfunction and insulin resistance, a key feature of type 2 diabetes. Maternal undernutrition including the dietary restriction of folate and methionine which are methyl-group providing nutrients have been shown to lead to DNA-methylation changes in genes controlling glucose and insulin homeostasis in the offspring. IUGR resulting from maternal undernutrition has been shown to lead to hypermethylation and silencing of key genes such as Pdx1 (the transcription factor required for beta-cell function) and glucokinase leading to a decrease in insulin secretion potential and increasing the risk of developing type 2 diabetes later in life (64, 65).

Maternal undernutrition affects the imprinting of genes such as IGF2 and affects growth and metabolism in offspring. Inappropriate gene imprinting can cause an epimutation through altered gene expression leading to enhanced IGF2/IGF1 signaling and increased glucose uptake leading to increased risk of developing diabetes (66–68).

High methylation of PPARGC1A gene resulted in downregulation of gene expression, leading to a deficiency in insulin secretion and consequently type 2 diabetes (69). Abnormal histone modification was also proposed to cause type 2 diabetes through histone deacetylases (HDACs) mediated suppression of GLUT4 gene expression in adipocytes, thereby causing insulin resistance (70, 71). Effects of various dietary components on miRNAs that are directly and indirectly involved in the modulation of the insulin signaling pathway and the regulation of pancreatic beta-cell function were investigated. Adipose tissue-specific miR-143 and pancreatic beta-cells specific miR-375 were reported to be involved in insulin secretion and glucose metabolism (70, 71).

## 4 Mechanistic insights of malnutrition induced β-cell dysfunction: nutrient-mTOR-PDX1-pancreatic β cell function-type 5 diabetes axis

**Figure 3:**
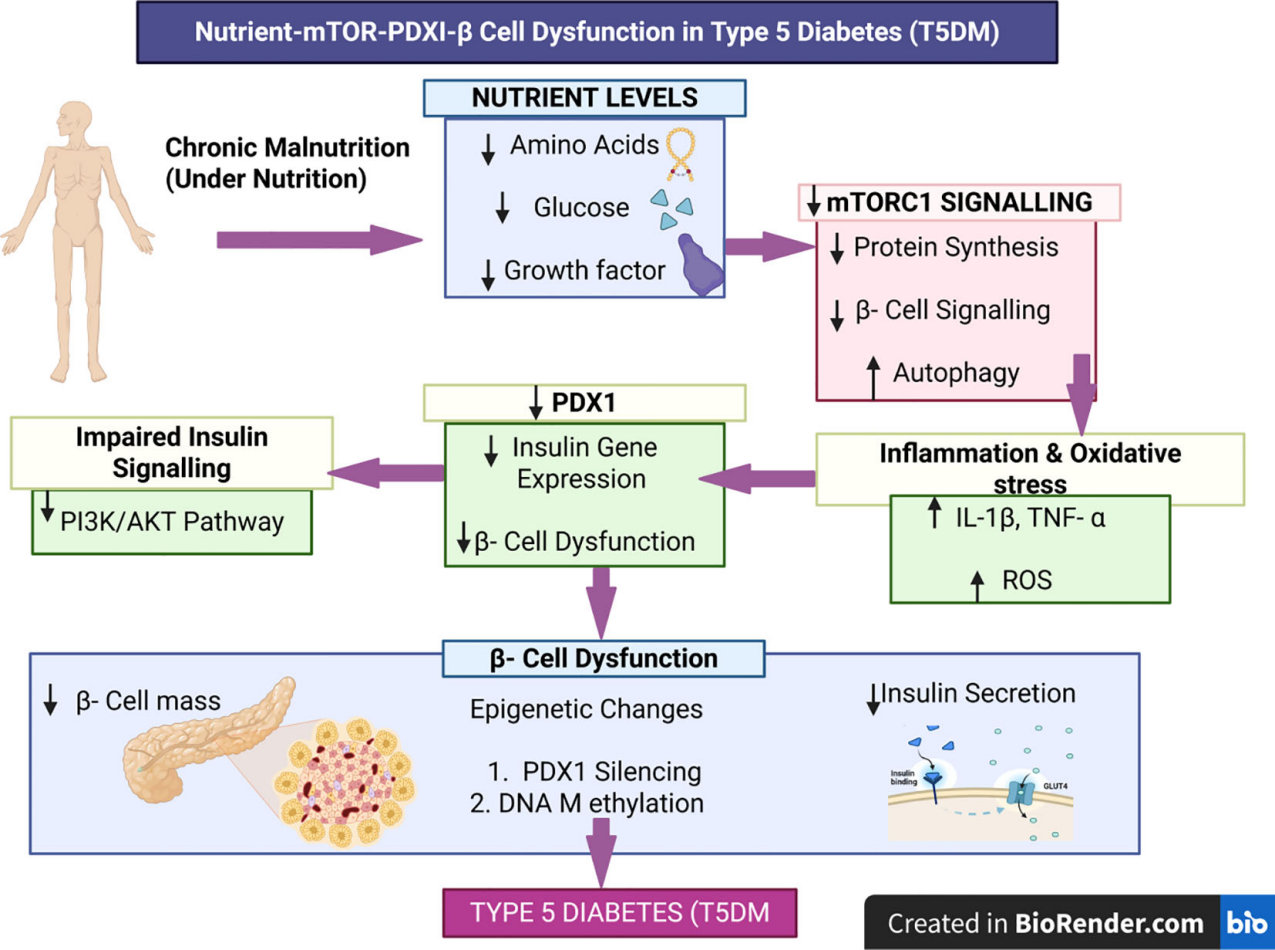
Nutrient–mTOR–PDX1-axis in β-cell dysfunction underlying type 5 diabetes mellitus (T5 DM). Illustrates Schematic illustration of how chronic malnutrition leads to β-cell dysfunction in Type 5 Diabetes Mellitus. Chronic undernutrition creates a “caloric poor” environment that suppresses availability of essential nutrients including amino acids, glucose and growth factors that activate mTORC1. Down-regulated mTORC1 leads to decreased protein synthesis and increased autophagy. PDX1, a transcription factor essential for insulin gene expression, is suppressed. Malnutrition triggers low-grade inflammation and oxidative stress by increasing production of pro-inflammatory cytokines (e.g. IL-1β, TNF-α) and reactive oxygen species. We also demonstrated impaired insulin signaling via the PI3K/AKT pathway. The combined effects of malnutrition and altered insulin action resulted in reduced beta cell mass, changes in gene expression involving epigenetic silencing of PDX1 gene via demethylation of its promoter region, and impaired insulin secretion. These mechanisms resulted in the development and progression of Type 5 Diabetes Mellitus.

Malnutrition-associated diabetes, also known as Type 5 diabetes mellitus (T5DM), is primarily caused by the gradual dysfunction and loss of β-cells due to prolonged nutrient deprivation and the disruption of precisely controlled nutrient-sensing pathways. In contrast to type 2 diabetes, which is mainly characterized by insulin resistance, T5DM involves a fundamental issue with β-cell mass, insulin production, and adaptive capacity, all stemming from nutritional deficiencies experienced early in life and persisting over time. The nutrient–mTOR–PDX1–β-cell axis serves as a comprehensive mechanistic framework that connects external nutrient availability with internal signaling, transcriptional regulation, and cellular metabolism (**Figure 3**).

### 4.1 Nutrient sensing and mTORC1 dysregulation

The mTORC1 pathway acts as a key metabolic sensor, integrating signals from amino acids (especially leucine), glucose, and growth factors like insulin and IGF-1 to control the growth, proliferation, and protein synthesis of pancreatic β-cells. Under normal physiological conditions, mTORC1 activation facilitates ribosomal biogenesis and insulin translation, boosts mitochondrial metabolism and ATP production, and aids cell cycle progression through cyclin activation and PDK1 signaling. However, during chronic malnutrition, the limited availability of essential nutrients and energy substrates leads to a sustained suppression of mTORC1 activity. This suppression results in hindered β-cell proliferation and regeneration, reduced insulin biosynthesis, and impaired mitochondrial function with decreased ATP production. Additionally, experimental evidence suggests that the loss of mTOR signaling in β-cells triggers oxidative stress, elevates thioredoxin-interacting protein (TXNIP) expression, and activates cellular stress pathways, ultimately leading to β-cell dysfunction and apoptosis (72, 73).

### 4.2 PDX1 role in transcriptional regulation and β-cell dysfunction

The influence of mTORC1 signaling is significantly mediated by PDX1, a crucial transcription factor necessary for insulin gene transcription, β-cell differentiation and maturation, and the preservation of β-cell identity. mTORC1 influences PDX1 through various mechanisms, including protein stability, phosphorylation, and transcriptional activity. Recent findings (74) indicate that mTORC1-dependent phosphorylation boosts PDX1’s function, thereby enhancing insulin production and β-cell proliferation. However, in situations of prolonged malnutrition, diminished mTOR activity results in lower PDX1 expression and functionality. This is further exacerbated by epigenetic silencing mechanisms such as DNA methylation and histone modifications, along with microRNA-mediated suppression of PDX1 and its related signaling pathways. As a result, there is impaired insulin gene transcription, defective glucose sensing, and a gradual loss of β-cell phenotype, which are characteristic features of Type 5 diabetes mellitus (T5DM).

### 4.3 Impaired insulin signaling pathways

Nutrient scarcity also disrupts the upstream insulin and IGF-1 signaling pathways, especially the PI3K–AKT axis, which typically activates mTORC1 and aids in the survival and function of β-cells. Weakening of this pathway leads to reduced phosphorylation of essential downstream targets like FOXO1 and GSK3β, resulting in impaired glucose-stimulated insulin secretion (GSIS) and heightened vulnerability to apoptosis. Crucially, this creates a harmful feedback loop, where impaired insulin signaling further diminishes mTOR activity, thereby worsening β-cell dysfunction and metabolic failure.

### 4.4 Inflammatory and immune microenvironment

Although T5DM is not traditionally considered an autoimmune condition, persistent malnutrition significantly disrupts immune balance, leading to low-grade inflammation and heightened oxidative stress in the pancreatic microenvironment. Increased levels of pro-inflammatory cytokines like IL-1β and TNF-α contribute to β-cell damage by activating stress signaling pathways and producing reactive oxygen species (ROS). Recent findings suggest that localized inflammatory niches within pancreatic tissue further undermine β-cell survival and function. These disturbances in the microenvironment interact with nutrient-sensing pathways, intensifying metabolic stress and hastening β-cell loss (73, 75).

### 4.5 Autophagy metabolism imbalance and mitochondrial dysfunction

mTORC1 serves as a crucial inhibitor of autophagy, a vital process for preserving cellular equilibrium. Under typical physiological circumstances, the balanced activity of mTOR ensures that autophagy levels are optimal, supporting both insulin secretion and cellular integrity. However, prolonged malnutrition results in the continuous suppression of mTORC1, leading to an overactivation of autophagy. Although basal autophagy is protective, its excessive activation leads to the breakdown of essential cellular components, mitochondrial dysfunction, and a decrease in insulin granule formation. Impaired mitochondria further reduce ATP production, which is essential for insulin secretion, thereby contributing to the gradual failure of β-cells (75).

### 4.6 Epigenetic and developmental programming

Malnutrition experienced early in life, especially during fetal development and when intrauterine growth is restricted (IUGR), leads to enduring epigenetic changes in pancreatic β-cells. These changes encompass DNA methylation of crucial regulatory genes like PDX1 (74), modifications to histones that affect chromatin accessibility, and the suppression of elements in the mTOR signaling pathway by microRNAs. These epigenetic shifts cause lasting impairments in the mass, proliferation ability, and functional capacity of β-cells, increasing the likelihood of developing T5DM in later life.

Persistent malnutrition results in decreased nutrient availability, which in turn inhibits mTORC1 activation and subsequently reduces PDX1 activity. This chain of events leads to compromised insulin production and a decrease in β-cell mass, ultimately causing β-cell dysfunction and the onset of T5DM. This central pathway is further influenced by disrupted insulin signaling, inflammatory and oxidative stress pathways, dysregulated autophagy, mitochondrial dysfunction, and epigenetic changes, all of which collectively drive the progression of the disease. The nutrient–mTORC1–PDX1–β-cell pathway offers a detailed mechanistic framework that connects chronic undernutrition to β-cell dysfunction in T5DM. Unlike other diabetes types, this model focuses on the nutrient-induced failure of β-cell adaptation rather than autoimmune destruction or primary insulin resistance. This distinction underscores unique therapeutic possibilities, where strategies targeting nutritional rehabilitation, mTOR signaling modulation, and reversal of epigenetic changes may provide effective approaches for preventing and managing malnutrition-related diabetes.

## 5 Emerging evidences

The proposed contributing factors to this sort of diabetes encompass antioxidant insufficiency, chronic undernutrition, and epigenetic alterations (76, 77). Significantly, research from Jamaica indicates that individuals aged 17 to 50 who have recovered from marasmus demonstrate less insulin secretion and heightened glucose intolerance relative to those who have recovered from kwashiorkor, in comparison to control patients (78). Autopsies of stillborn fetuses from women with anemia have revealed a notable decrease in the percentage of beta cells compared to those from moms with normal hemoglobin levels throughout pregnancy. Additionally, reprogramming of the fetal pancreatic islets was noted, with an increase in alpha cells by up to 20% compared to non-anemic mothers, an elevation in non-alpha/beta cells, and a modification in the alpha to beta cell ratio (79). Research identified diminished beta cells in the pancreas of children who succumbed to protein-energy deficiency. Early malnutrition or intrauterine growth restriction (IUGR) epigenetically modifies PDX1, thereby affecting β cell proliferation and insulin gene expression, which impairs β cell responsiveness in adulthood. Cellular replication, particularly of b-cells, was diminished by about 50% in the low-protein progeny, attributed to an extended cell cycle and elevated cyclin D1, a marker of the G1 phase, whereas NEK2, an indicator of cells in G2 and mitosis, was reduced. This may also arise from the persistent programming of cell kinetics that could have been imprinted on precursor cell populations prior to the onset of endocrine differentiation (80). The population of endocrine precursor cells that tested positive for markers such as nestin, CD34, and c-Kit diminished over the perinatal period. Accelerated apoptosis has been documented, which may contribute to a diminished endocrine cell mass. A reduced quantity of mitochondria in leukocytes may be associated with low birth weight and diminished glucose tolerance in adults. Research in both animals and people has underscored the significant impact of early feeding on lipid metabolism (81).

Animal experiments validate this concept, demonstrating that a prolonged low-protein diet markedly diminishes both baseline and post-glucose-induced insulin production in comparison to mice on a standard diet. A study found that mice with protein malnutrition, devoid of sepsis or other inflammatory conditions, displayed decreased insulin secretion, glucose intolerance, hepatic dysfunction, and impaired hepatic clearance. These traits are comparable to the pathophysiological foundation of Type 5 diabetes mellitus in individuals experiencing chronic starvation from childhood into adulthood (82).

The exact mechanisms underlying defects in insulin secretion remain unclear; however, deficiencies in body potassium and/or chromium, reduced gastrointestinal absorption, impaired gut insulinotropic factors, and dysfunctional insulin transport by the pancreas have all been cited and are corroborated by multiple studies (83). Additionally, animals suffering from chronic anemia throughout fetal development have been observed to demonstrate glucose intolerance. This was evidenced in fetal sheep, when anemia was generated, resulting in glucose intolerance in comparison to healthy sheep without anemia. The situation was ameliorated by enhancing oxygenation and addressing the anemia. This indicates that postnatal undernutrition may lead to glucose intolerance (84). Research showed that lean BMI diabetes mellitus in a cohort resembling Type 5 diabetes exhibits immunogenetic distinctions from Type 1 diabetes (85).

Evidence indicates that dysfunction of the incretin axis, crucial for glycemic homeostasis and potentially compromised by starvation or early life malnutrition, adversely affects gut incretin response. This, in turn, modulates the expression of signaling molecules such as PAK1 and β-catenin, which are responsible for GLP-1 expression and its subsequent secretion from L cells into the bloodstream (86).

The Barker hypothesis posits that type 2 diabetes mellitus originates in individuals with a lean BMI at birth, then develop the condition due to excessive dietary intake during infancy and adolescence, potentially leading to an increased risk of cardiovascular abnormalities (87). They anticipate a scenario in which undernutrition or placental insufficiency arises during pregnancy, succeeded by postnatal undernutrition, resulting in diminished beta cell mass, reduced muscle mass, and wasting (88). This encapsulates the fundamental nature of Type 5 Diabetes. Nonetheless, the specific molecular connections between hunger and diabetes require more clarification. The moment has arrived to explore this link further, as comprehending it could transform our strategies for preventing and managing diabetes.

High-resolution mapping of the architecture of pancreatic tissue while maintaining spatial context has been made possible by recent developments in spatial omics technology. Significant intra-islet variation in β-cell transcriptional programs, stress signaling pathways, and endocrine cell neighborhood structure has been shown using spatial transcriptomics and multiplexed imaging techniques (89). Spatial transcriptome analysis in the human diabetic pancreas has led to the enrichment of inflammatory and oxidative stress signatures in peri-islet niches and the region-specific inhibition of β-cell identity genes (**Figure 2**) such PDX1, MAFA, and NKX6.1 (89, 90).

## 6 Overview of spatial omics technologies

Spatial omics technologies enable the simultaneous detection and localization of molecular constituents within tissues, providing unparalleled insights into cellular and tissue architecture at high spatial resolution. These advancements primarily fall into two categories: imaging-based approaches and sequencing-based methods. Imaging-based techniques, derived from single-molecule hybridization strategies, allow high-resolution visualization of targeted molecules, transitioning from single-gene detection to multiplexed profiling of hundreds to thousands of transcripts. Sequencing-based methods, on the other hand, facilitate unbiased, genome-wide analysis with progressively improved spatial resolution, from regional to subcellular levels (91).

### 6.1 *In situ* imaging-based spatial omics

Imaging-based approaches typically involve two steps; designing nucleic acid probes such as fluorescent or sequence-tagged oligonucleotides that hybridize to target transcripts; and capturing images through fluorescence microscopy. Techniques like smFISH (single-molecule fluorescence *in situ* hybridization) exemplify early spatial transcriptomics (92), providing single-molecule resolution but limited multiplexing capacity (93). Advances such as MERFISH, seqFISH, and seqFISH+ have expanded gene detection into thousands of transcripts by employing combinatorial barcoding, iterative hybridization, and error-robust encoding schemes. While highly sensitive and capable of single-molecule detection, these methods are often labor-intensive and costly due to multiple hybridization cycles and probe synthesis requirements. Expansion microscopy further enhances resolution and detection efficiency by physically enlarging samples, enabling more detailed subcellular analysis.

### 6.2 Sequencing-based spatial omics

Sequencing-based approaches capture spatial gene expression profiles through *in situ* capture and subsequent sequencing. Early methods like Tomo-seq and Geo-seq provided regional resolution but were limited by physical sectioning. Recent innovations, including Slide-seq, HDST, and Visium, utilize barcoded beads or microfluidic devices to achieve resolution from tens of micrometers down to subcellular levels. Techniques such as spatial transcriptomics (ST), NICHE-seq, and microfluidic-based methods like DBiT-seq and Slide-seqV2 have pushed resolution further, approaching cellular and even subcellular scales. Advancements in microfluidics and nano-array chips such as Stereo-seq and Pixel-seq have achieved resolutions as fine as 500 nanometers, enabling precise mapping of cellular and molecular landscapes (94, 95).

### 6.3 Non-transcriptomic spatial omics

While spatial transcriptomics has greatly advanced our understanding of gene expression within tissues, it only represents one aspect of the complex molecular landscape (96). Other omics layerssuch as genomics, epigenomics, translatomics, proteomics, and metabolomics provide additional insights into cellular functions and states, offering a more comprehensive view of biological processes and disease mechanisms.

Spatial Genomics focuses on analyzing DNA variations within tissues. Technologies like *slide-DNA-seq* enable direct sequencing of DNA from fixed tissue sections, capturing spatial information about genetic differences, such as tumor clones (97). This approach is valuable in studying tumor evolution and heterogeneity, especially in cancer research, by mapping DNA copy-number alterations across tissue regions.

Spatial Epigenomics involves techniques like *spatial ATAC-seq* and *spatial CUT&Tag*. These methods map chromatin accessibility and histone modifications within tissues, revealing how epigenetic regulation varies across different cell types and regions (98). Such insights help understand gene regulation mechanisms in development and disease at the spatial level.

Spatial Proteomics utilizes methods such as CODEX (91) and *Immuno-SABER* that use DNA-barcoded antibodies to detect multiple proteins simultaneously within tissue sections. Advanced techniques like *single-cell deep visual proteomics (scDVP)* combine high-content imaging with mass spectrometry, enabling detailed analysis of protein expression at single-cell resolution within the tissue context (99). These approaches clarify protein interactions and functions critical in cellular processes and pathology. Spatial Translatomics examines the process of protein synthesis by locating ribosomes and translating mRNA within tissues. Techniques like *RIBOmap* offer insights into how proteins are produced in specific cellular and subcellular regions, enriching our understanding of gene expression regulation at the translational level in tissue architecture. Spatial Metabolomics maps the distribution of metabolites within tissues, linking metabolic activity directly to tissue function (100). Techniques such as *mass spectrometry imaging (MSI)* and *scSpaMet* visualize and quantify metabolites with high spatial resolution, providing valuable information on metabolic pathways involved in health and disease (101). Together, these non-transcriptomic spatial omics layers complement transcriptomics by revealing different molecular mechanisms operating within tissues, thereby advancing our understanding of cellular behavior, tissue organization, and disease processes.

### 6.4 Spatial multi-omics

Spatial multi-omics integrates multiple molecular layers within the same tissue context, providing a comprehensive view of cellular function and regulation. Techniques like *CITE-seq* and *Stereo-CITE-seq* combine transcriptomics and proteomics by using DNA-barcoded antibodies alongside RNA sequencing, enabling simultaneous measurement of gene expression and protein levels at spatial resolution. This reveals detailed cellular phenotypes and interactions.

Other approaches, such as *spatial ATAC-seq* combined with RNA-seq, map chromatin accessibility and gene expression together, offering insights into epigenetic regulation and transcriptional activity within the same cells. Similarly, *CUT&Tag-RNA-seq* merges epigenomic profiling of histone modifications with transcriptomics, illuminating how epigenetic changes influence gene expression dynamically (102). These integrated methods deepen our understanding of the regulatory mechanisms governing cell behavior during development and disease.

### 6.5 Temporally resolved spatial omics

Understanding how biological processes change over time is important. Techniques like single-cell omics, lineage tracing, metabolic labeling, molecular recording, and *in vivo* fluorescent labeling help researchers study cell development and disease at the single-cell level (103). Many biological and cellular processes have been studied at single time points in single conditions using omics approaches. However, almost none have been studied in a continuous fashion. Recent developments in spatial omics have incorporated DNA barcodes to link temporal information. An example of such approaches was demonstrated in intMEMOIR, where integrase enzymes encoded in DNA barcodes (cellular markers) are used to record lineage information. The approach involves introducing mutations into DNA sequences that are introduced into cells and are modified or mutated over time to record the events that have occurred. The barcodes can then be imaged over time to visualize cellular development processes. Another example is spatial iTracer, where reporter barcodes are fused with CRISPR-Cas9 to generate scars at specific time points in the genome that can be readout via sequencing to follow the lineage of cells and to study cell decision-making processes such as differentiation. Another approach called Space-TREX uses a lentiviral approach to introduce unique cell-inherited DNA barcodes into cells of interest (such as in mouse brain tissue) while providing the unprecedented capability of coupling single cell transcriptomics and spatial transcriptomics to elucidate relationships between cells. However, the use of these approaches is currently limited by the need to improve sensitivity and precision of the detection of the DNA barcodes as well as to improve the resolution and accuracy of the imaging of these markers in order to obtain reliable data with wider applicability (104).

### 6.6 Data analysis tools for spatial omics

Spatial omics offers a unique opportunity to gain new insights into the tissue structure and function and analyzing the resulting data often requires advanced bioinformatics methods. While single cell omics provides the information from each individual cell, the spatial information from spatial omics allows us to locate with high precision the position of the cells in space, and from the resulting tissue maps we can obtain detailed information on tissue architecture at different scales at the level of cell density, spatial distribution and cell to cell communication, which reflect the morphology of the cells; by decomposing multi-cellular regions into sub-compartments, which is especially relevant for understanding the complexity and heterogeneity of biological samples; at the single cell level and across multiple biological scales, from the molecular to the organ/tissue level, in case of high resolution data.

Most analysis workflows involve some form of data preprocessing to prepare the data for further analysis. After that, segmentation of the spatial regions in the tissue of interest is needed. For cellular-scale datasets, cell type classification is achieved through either alignment of cells to known reference profiles or through clustering of cells into classes. Analysis of spatial features such as genes with altered expression, cell and subcellular localization and protein-protein interactions (PPIs) generally yield the most information content. This can give a deeper understanding of how cells behave within the context of the tissue and how cells contribute to tissue behavior. As many of the recent review articles on single-cell omics highlight, they focus on available tools and challenges in the field. Several ongoing research efforts are aimed at developing new and improved methods at all biological scales, including the molecular, cellular, tissue, 3D and in time (105).

Spatial omics can be applied at the molecular level to analyze where molecules are located. Furthermore, the technology can be used to determine genes that show differences in expression levels in various sub-regions within a tissue, so called spatially variable genes (SVGs), and thus obtain information about tissue compartments and tissue functions (106). Insights into gene sets and regulatory networks can also reveal the complex regulatory mechanisms that ensure that genes are expressed at the right time in the right place within the spatial structure of a tissue. Finally, where the transcripts of certain genes are not detected, the expression values can be imputed (i.e., predicted) to maximize the quality of the resulting spatial gene expression data, e.g. by integrating the information with single-cell data that can be overlaid to the spatial landscape, or by computationally predicting the gene expression values at every location in the spatial landscape, which is often a highly computationally demanding process involving sophisticated statistical analysis.

Single cells are segmented and annotated at the cellular and subcellular level. In general, cells are usually detected at the imaging level on stained images. However, nucleus bias often appears in images of high density cell culture, and *in situ* sequencing approaches combining imaging and sequencing information also leads to inaccurate alignment of the molecular signal to the cell boundary. In addition, deriving neighborhood composition features to describe the similarities among locations and among cells is also a popular strategy, but often encounters the problems of sparse data and noise (107). The transformer architecture is more recent research direction for this research area.

All seeable multi-cellular resolution data needs cellular type specific markers for deconvolution and at best can give a best guess of the composition of the tissue at hand. Deconvolution does not always work well to rare or heterogeneous populations in a tissue. Also, region level analysis can provide better information than spot level analysis and can give an initial insight into the organization of the tissue at the level of specific tissue regions with different cellular composition and underlying gene expression and cell to cell contact. Although the analysis of graph neural networks and tissue images can improve these analyzes, they are still data limited and thus prone to overfitting. More studies are exploring cell–cell communication beyond the proximity of cells including ligand-receptor networks and dependency models to empirically assess the dependency between the expression of genes in one cell and in adjacent cells (108).

3D reconstruction of tissues in 3D gives a better view of the tissue structure. Using image analysis software such as CODA, PASTE and SPACEL enables us to stack together slices of data to form a 3D reconstruction that allows us to better visualize the cellular interactions that occur within the tissue and the structure of the tissue. 3D reconstruction is an important tool to study how tissues and cells develop in the microgravity of space and over time. In addition, reconstructing a 3D view of a tissue is very important for studying organ development from embryonic origins and understanding tissue homeostasis by combining lineage tracing with cell trajectory analysis (109).

Spatial omics has led to a large amount of online data repository, where data could be deposited and shared. Initially, these resources were based on model organisms and imaging data. Now we have single-cell resources like Human Cell Atlas and HuBMAP. There are many data resources for spatial transcriptomics data resources, such as STOmicsDB (110–114) and SpatialDB (115) and SOAR (116). These resources have been successfully applied in many studies like cancer (117), neuroscience (118) and so on. In the future, the resources should be more integrated and multi-omics, including proteomics, metabolomics and imaging data. Therefore, the data resources should be improved to facilitate the analysis of omics data and to find valuable knowledge in an efficient way.

In brief, spatial omics is a new way of studying how tissues behave and how they develop. By making the molecular signals in the tissue visible, we can get a better understanding of where genes are expressed in the tissue and where the different cell types are located. This knowledge of the tissue architecture can provide crucial information about the cellular microenvironment and how it influences the behavior of the tissue. Furthermore, by studying organ function, organ development and disease at a higher resolution, we can identify better biomarkers and treatment strategies.

The Human Protein Atlas has undergone many developments during the years and has now reached a level where it can be utilized to a larger extent in both research and medicine. Despite the developments that have taken place, there is still work to be done in order to be able to utilize the information in its full potential. The majority of present day research is based on studies of gene expression and little is still investigated within the field of epigenomics and proteomics. Continued multi-omics research as well as further development of high-resolution 3D atlases of the human body will lead to further knowledge regarding tissue structure and biology, which in turn will generate new information. This in turn will open novel opportunities for future treatment and finally will lead to precision medicine (119).

## 7 Integrated spatial multi-omics for high-resolution mapping of tissue microenvironments

Integration of different spatial omics technologies allows for understanding of spatial organization of biological tissues and for discovery of biomarkers within specific tissue contexts. Spatial multi-omics methods, such as CINDEL, MGIX and CHiT-Seq technologies, enable simultaneous analysis of gene expression study across whole tissues, without cell dissociation, to yield average signals reflecting tissue-wide dynamics, and to elucidate tissue-wide structural and functional organization. Here, we globally map nutrient-sensing gene expression as well as β cells and their micro-environment across the pancreatic and gastrointestinal tissues from Type 5 diabetes mellitus (T5DM) induced by malnutrition. The disease primarily affects β cells.

Fixed tissue sections of pancreas and gut were used for high-resolution tissue imaging, allowing for optimal fixation preserving the intricacies of tissue structure and including the mucosal surface exposed to microbiota in intestinal tissue. High resolution images of H&E stained sections and immumofluorescence staining allowed for clear definition of islets, epithelial tissue, stroma and immune infiltrates within tissues. Spatial omics technologies that provide multi-layered data with spatial information including spatial transcriptomics, spatial proteomics by MIBI and spatial metabolomics by MALDI-MSI were registered to high resolution histology images of sections. The registration of data was performed by Seurat and Giotto packages.

Cell segmentation and annotation (such as cell classification into biologically relevant cell types like β-cells, α-cells, immune cells, stromal cells etc.) and subsequent spatial mapping of these cell populations within a tissue is an important component of the tissue microarchitecture mapping pipeline. We observe that T5DM induced changes in the pancreas are not uniform across all islets. Some islets are significantly depleted of β-cells whereas other islets have become populated by immune cells and formation of clear inflammatory zones. The third category of islets show metabolic changes but within specific niches.

While descriptive mapping of single-cell data is very powerful for getting a handle on how different cellular populations are spatially arranged within a tissue, the next level of analysis is quantitative spatial analysis. Using specialized software such as Squidpy, paired comparisons between single cells can be formally performed to extract quantitative information about spatial relationships such as proximity, clustering and niche occupancy. Therefore, we can ask questions about the spatial organization of β-cells and whether they are alone, embedded within inflammatory cells or ‘starving’ within certain niches. We can also ask about the existence of functional tissue domains, such as intact functioning islets versus dysfunction or fibrosis. Furthermore, these niches can be dissected apart using multiple layers of information including cell type, gene expression and metabolism (e.g. an inflammatory niche versus nutrient deficiency).

While spatial organization of the tissue and cell–cell interactions can affect β-cell function, modeling these microenvironments can provide more insight into how these microenvironments control β-cell function and diabetes. Here, we describe how to use COMMOT to connect ligand–receptor gene expression data to information about spatial relationships between cells, to predict intercellular signaling networks. These connections reveal how immune cells, stromal cells or epithelial cells can control β-cell survival and insulin secretion. The connections can be visualized to map local cytokine signaling, for example IL-1β and TNF-α-induced β-cell stress, as well as nutrient-sensing signaling, for example mTOR signaling to metabolic gradients in the microenvironment.

Interrogating data from multiple omics platforms can provide greater understanding of tissue function by examining how different layers of gene expression (transcriptomics, proteomics and metabolomics) interact. Additionally, software such as Tangram can be used to integrate single cell data with spatial data to validate findings between these two platforms. In the case of T5DM, metabolism, mitochondria and nutrient signaling pathways all impact β-cell function. Therefore, integrating these platforms can provide novel insights into β-cell failure. We identified spatial co-localization of decreased PDX1 with increased mTOR pathway activity and metabolic stress in high-risk regions of pancreas.

Given the power of having both molecular and spatial information within a single biomarker, spatially resolved biomarkers (i.e. biomarkers that contain both molecular and spatial information, and are spatially resolved such as those obtained in T5DM shown to the left) are more powerful than bulk biomarkers. Some potential biomarkers in T5DM include insulin producing beta cells: β-cell–specific markers (e.g., reduced PDX1, insulin expression), Nutrient–sensing pathway markers (e.g., altered components of mTOR signaling), Inflammatory mediators localized to specific niches, Metabolic (Mt) profiles indicating either mitochondrial dysfunction or altered energy metabolism, Signals from the microbiome and GI tract tissues which affect host metabolism. These biomarkers are not only associated with disease, but are also markedly enriched in the relevant tissue location and associated cellular niche. This specificity, and potential clinical utility, was a major consideration in their selection.

Finally, all gene expression analyzes need to be statistically and computationally validated to ensure robust results. This includes testing for spatial autocorrelation as well as performing a gene set enrichment analysis (GSEA) on individual samples. For the unsupervised analysis of bulk samples, we performed a non-negative matrix factorization (cNMF) to decompose the samples into putative cell types and to quantify and decompose single cell samples for comparison. In addition, we performed a correlation-based network analysis for pairwise comparisons of samples. All analyzes were corrected for batch effects and results were validated across datasets for reproducibility (120–123).

Spatial multi-omics integration of T5DM connect tissue microarchitecture to underlying disease mechanisms and identified biomarkers and mechanistic features that are context-dependent. Spatial multi-omics integration of T5DM therefore transforms the study of this common disease from a bulk, descriptive level of analysis to quantitative, spatially resolved, hypothesis-driven studies that will be highly useful for early diagnosis and patient stratification and to guide the development of tissue-specific, targeted therapies.

Recent advances in spatial omics have enabled the organization of molecular changes in the tissue microenvironment to better understand diabetes molecular mechanisms that were previously viewed as uniformly distributed among individual cells. single-cell multiome and spatial transcriptomics performed on non-diabetic, autoantibody-positive and type 1 diabetes (T1D) pancreas tissues demonstrated novel mechanisms that underlie β-cell dysfunction in T1D (124). The spatially organized immune interactions contributed to the autoimmune destruction of the β cells. An enhanced antigen presentation by β cells at sites of immune–epithelial destruction was demonstrated, revealing local immune niches to further elucidate the pathogenic mechanisms that lead to T1D. Literature employing spatial transcriptomics to map the pancreatic tissue from individuals with type 2 diabetes (T2D). Instead of uniform insulin and glucagon expression in islets, T2D was associated with insulin and glucagon-producing β-cell failure that was spatially restricted and highly dependent on the surrounding tissue microenvironment. Spatial multi-omics analyzes have also been performed outside of the pancreas (125).

A recent study explored spatial organization of diabetic vascular complications, uncovering local inflammatory and metabolic events within different vascular compartments, such as activation of arterial smooth muscle cells that acquired macrophage-like or fibroblast-like phenotypes in certain segments of arteries (126).

A recent study performed spatial profiling of islet transplantation and revealed distinct immune cell infiltration patterns around grafts, contributing to graft rejection and emphasizing the tissue microenvironment in determining the transplant outcome. Observations relevant to diabetes are presented that illustrate that diabetes is more than a systemic metabolic disorder of the body; it involves organized collections of cells that interact with each other and their surroundings, that are heterogeneous, and that have organized gene regulatory networks that can be exploited for early diagnosis and for spatially organized therapies (127).

## 8 Management

The therapy of type 5 diabetes presently lacks substantial evidence-based guidelines owing to inadequate data. Individuals with type 5 diabetes may necessitate only minimal insulin or alternate interventions to promote insulin secretion for the successful management of hyperglycemia while preventing hypoglycemia. Although sulfonylureas may be beneficial, vigilance is required to avert hypoglycemia. DPP-4 inhibitors may be safe, although they might not adequately resolve insulin insufficiency. GLP-1 receptor agonists are typically inappropriate since their weight loss effects may exacerbate diseases such as sarcopenia. Despite metformin demonstrating some empirical success, especially in persons with a low BMI (defined as a BMI <18.5 kg/m²), no evidence confirms its efficacy in this population.

SGLT2 inhibitors are viable therapy options due to their mode of action; nevertheless, the resulting weight loss may be detrimental for undernourished individuals. Treatments for type 5 diabetes are predominantly empirical, highlighting the need for additional nutritional and therapeutic research in this field.

Alongside medicine, it is essential to address the nutritional requirements of people with type 5 diabetes. Nutritional rehabilitation and health education are essential for fostering a balanced diet that satisfies protein, energy, and micronutrient needs vital components for successful long-term treatment. Sustainable dietary interventions for patients from low socioeconomic backgrounds should emphasize locally available, culturally appropriate options, including energy-dense, protein-rich staple foods like lentils, legumes, oil-enriched cereals, and fortified grains, in conjunction with community health education initiatives. The intervention should seek to integrate with current public health nutrition initiatives such as midday meal programs, maternity and child nutrition assistance, or conditional cash transfers. Community-based care of ready-to-use therapeutic foods or home-fortified alternatives could effectively address severe nutritional deficits.

Furthermore, management guidelines must address the emotional, cultural, and educational obstacles encountered by patients and their families. Due to the unusual clinical manifestation of type 5 diabetes, it is imperative to inform the population about its diagnostic and therapeutic protocols, hence improving treatment compliance. Future research should concentrate on techniques that mix educational activities with psychosocial assistance to enhance health outcomes for those with type 5 diabetes.

## 9 Prevention

Educational initiatives designed to enhance nutrient consumption among pregnant women have demonstrated restricted efficacy, chiefly due to obstacles associated with food accessibility in high-burden environments. While these programs may mitigate the risks of preterm birth and low birth weight (LBW), their efficacy is frequently compromised in the absence of sufficient nutritional resources. For women in food-insecure settings, specifically designed nutritional items are vital.

Deficiencies in micronutrients are widespread among pregnant women in low- and middle-income countries (LMICs). Multiple micronutrient (MMN) supplements are a standardized collection of vital vitamins and minerals aimed at improving the nutritional condition of pregnant women. These supplements have demonstrated efficacy in mitigating the risks of low birth weight, preterm birth, and enhancing long-term health outcomes for children. The Women’s First Trial reveals that initiating supplementation prior to conception or in early pregnancy enhances outcomes; however, multiple micronutrient supplements alone cannot rectify undernutrition and should be combined with methods to augment macronutrient consumption.

During pregnancy, the intake of macronutrients, especially protein and energy, is essential, necessitating an increase of 21 grams of protein and 420 kilocalories of energy per day. The quality of protein is crucial, as numerous low- and middle-income countries depend on plant-based sources that are deficient in essential amino acids (EAAs), potentially resulting in nutritional inadequacies for mothers and fetuses. Balanced energy protein supplements (BEPs) recommended by the WHO, supplying roughly 25% of energy from protein, are associated with decreased incidences of stillbirths, low birth weight (LBW), and small-for-gestational-age (SGA) newborns. Numerous BEP products are available, such as fortified biscuits, beverages, and lipid-based nutrient supplements (LNS), which can fulfill the nutritional requirements of pregnant women.

Lipid-based nutrient supplements, customizable to address specific micronutrient requirements, have demonstrated efficacy in promoting infant weight and length while reducing the risks of low birth weight and stunting, but their effectiveness may fluctuate depending on the nutritional state of the pregnant woman.

## 10 Future perspectives

The discourse on type 5 diabetes is grounded in the current knowledge with lean individuals experiencing undernutrition and relevant animal models. This diabetic phenotype necessitates comprehensive further investigation to elucidate its etiology, impact, and natural progression. Furthermore, longitudinal studies are crucial for comprehending the lifelong effects of undernutrition on diabetes risk and the potentially complicated pathophysiology of this diabetes type. Creating a global registry could enhance such studies. International research collaborations aimed at assessing the diagnostic criteria for this type of diabetes across many populations would enhance the definition of this phenotype.

The implementation of diagnostic tools should, at a minimum, involve a fasting C-peptide level to confirm definitive beta cell insufficiency, and in more resource-rich environments, a postprandial C-peptide level as well. At a minimum, an anti-GAD (glutamic acid decarboxylase) antibody test is necessary to exclude Type 1 diabetes, however many resource-limited settings may find it challenging to do this test, let alone the complete array of tests.

An abdominal ultrasound, or preferably a CT scan of the abdomen, is necessary to exclude pancreatic diabetes. Ultimately, interventional studies are essential to develop evidence-based care guidelines for doctors treating patients with type 5 diabetes. The Vellore Declaration urges the global diabetes community to officially acknowledge this overlooked condition, which probably impacts the quality and duration of life for millions globally. We urge international organizations, including the IDF and WHO, to advocate for further study on the phenotype, etiology, and treatment of type 5 diabetes. Additional investigations involving both retrospective databases and clinical interventional trials concerning food, exercise, and medicinal drugs are crucial in this field. In conclusion, Type 5 diabetes represents a unique, non-autoimmune, lean diabetes phenotype with distinct pathophysiology and clinical features compared to Type 1 and Type 2 diabetes, as summarized in (**Table 1**).

Further evidence of changes in α:β cell spatial ratios and remodeling of islet cytoarchitecture under metabolic stress conditions is provided by multiplexed ion beam imaging and imaging mass cytometry investigations. Crucially, integration of single-cell and spatial multi-omics has shown defective insulin granule formation, endoplasmic reticulum stress signaling, and mitochondrial dysfunction pathways that are exclusive to β-cell clusters exposed to inflammatory microenvironments. The use of spatially resolved molecular profiling in Type 5 diabetes may help determine whether developmental nutritional programming causes niche-specific β-cell vulnerability rather than uniform endocrine loss, given the documented β-cell depletion, altered α:β ratio, and epigenetic PDX1 silencing seen in malnutrition-associated diabetes.

## 11 Finding evidence-based treatment plans

The absence of a tailored treatment strategy not only undermines day-to-day management but also increases the risk of early disability or premature death. Poor glycemic control accelerates complications such as retinopathy, nerve damage and kidney disease consequences that could be mitigated with the correct diagnosis and care.

Given its unique pathophysiology, standard treatments for type 1 or type 2 diabetes may not work. Research suggests that therapies that improve insulin secretion and nutritional status could provide better health outcomes. However, for now, further studies are needed to establish evidence-based treatment plans.

## 12 IDF type 5 diabetes working group

With the recognition of type 5 diabetes comes the need to develop specific treatment guidelines. Together with this recognition at the IDF World Diabetes Congress 2025 came the announcement of a new IDF working group, the Type 5 Diabetes Working Group, chaired by Dr Meredith Hawkins of the Albert Einstein College of Medicine and co-chaired by Dr Nihal Thomas of Christian Medical College in India. This group will draft formal diagnostic criteria and therapeutic guidelines over the next two years. Their aims include:

- Establishing clear diagnostic criteria to differentiate type 5 diabetes from other forms.
- Creating a global research registry to collect and analyze patient data.
- Developing educational modules to train healthcare professionals, particularly in low- and middle-income countries (LMICs).

This IDF Working Group paves the way for much-needed progress: the development of accurate diagnostic criteria, evidence-based treatment strategies and context-specific education for healthcare providers.

By detecting tissue-level molecular fingerprints as opposed to only circulating markers, emerging spatial omics systems provide new biomarker options. Within certain islet areas, spatial transcriptome mapping of the diabetic pancreas has revealed activation of NF-κB-mediated inflammatory pathways, elevated expression of dedifferentiation markers, and localized inhibition of β-cell maturation genes (PDX1, MAFA). Additionally, altered spatial distribution of insulin, proinsulin, and mitochondrial proteins have been found by imaging-based proteomic investigations, indicating that β-cell dysfunction may occur before overt cell death. Furthermore, the idea of epigenetically imprinted metabolic susceptibility is supported by integrated spatial epigenomic investigations that show region-specific DNA methylation alterations in genes controlling mitochondrial biogenesis (PPARGC1A) and glucose sensing (**Figure 2**). Spatially resolved β-cell gene expression profiles and mitochondrial density indicators may function as molecular diagnostic hallmarks that differentiate Type 5 diabetes from Type 1 and Type 2 diabetes in the setting of early-life starvation driving epigenetic remodeling.

## 13 Conclusion

This long-overdue official recognition of type 5 diabetes enriches our understanding of the broader diabetes landscape and the health disparities within it. By acknowledging the distinct pathophysiology of type 5 diabetes and the social determinants driving its emergence, the medical community is taking up the interrupted journey toward addressing a long-neglected condition affecting millions in under-resourced regions. Formal diagnostic criteria, targeted treatment strategies and region-specific education programs will contribute to eliminating misdiagnosis, improving health outcomes for millions and strengthening healthcare systems. As we anticipate rising diabetes rates, integrating type 5 diabetes into the global diabetes agenda is not only overdue, it is imperative.

The advent of spatial omics technologies marks a transformative leap forward, enabling precise mapping of microbiota-host interactions within their spatial and molecular contexts. These advancements hold great promise for unraveling complex disease mechanisms and developing targeted therapies. Looking ahead, future research will likely focus on integrating multi-omics spatial approaches, establishing causal relationships, and translating these insights into clinical applications, ultimately paving the way for personalized interventions in metabolic diseases.

## References

[1] Lontchi-Yimagou E Dasgupta R Anoop S . An atypical form of diabetes among individuals with low BMI. Diabetes Care. (2022) 45:1428–37. doi: 10.2337/dc21-1957. PMID: 35522035 PMC9184261

[2] Hugh-Jones P . Diabetes in Jamaica. Lancet. (1955) 2:891–7. doi: 10.1016/s0140-6736(55)92530-7. PMID: 13264638

[3] Kibirige D Sekitoleko I Lumu W . Phenotypic characterization of non-autoimmune diabetes in adult Ugandans with low body mass index. Ther Adv Endocrinol Metab. (2024) 15:20420188241252314. doi: 10.1177/20420188241252314. PMID: 38808009 PMC11131405

[4] Seiglie JA Marcus ME Ebert C . Diabetes prevalence and its relationship with education, wealth, and BMI in 29 low- and middle-income countries. Diabetes Care. (2020) 43:767–75. doi: 10.2337/dc19-1782. PMID: 32051243 PMC7085810

[5] Zhou B Rayner AW Gregg EW . Worldwide trends in diabetes prevalence and treatment from 1990 to 2022. Lancet. (2024) 404:2077–93. doi: 10.1016/s0140-6736(24)02317-1. PMID: 39549716 PMC7616842

[6] Kim JM Joung KH Kim HJ . Lean diabetes: 20-year trends in prevalence and clinical features among Korean adults. BMC Public Health. (2024) 24:3554. doi: 10.1186/s12889-024-21034-2. PMID: 39707280 PMC11662422

[7] Wadivkar P Jebasingh F Thomas N . Classifying a distinct form of diabetes in lean individuals with a history of undernutrition. Lancet Glob Health. (2025) 13:e1771–6. doi: 10.1016/s2214-109x(25)00263-3. PMID: 40975084

[8] Segerstolpe Å Palasantza A Eliasson P . Single-cell transcriptome profiling of human pancreatic islets in health and type 2 diabetes. Cell Metab. (2016) 24:593–607. doi: 10.1016/j.cmet.2016.08.020. PMID: 27667667 PMC5069352

[9] Baron M Veres A Wolock SL . A single-cell transcriptomic map of the human and mouse pancreas reveals inter- and intra-cell population structure. Cell Syst. (2016) 3:346–360.e4. doi: 10.1016/j.cels.2016.08.011. PMID: 27667365 PMC5228327

[10] Tang F Barbacioru C Wang Y . mRNA-seq whole-transcriptome analysis of a single cell. Nat Methods. (2009) 6:377–82. doi: 10.1038/nmeth.1315. PMID: 19349980

[11] Stuart T Satija R . Integrative single-cell analysis. Nat Rev Genet. (2019) 20:257–72. doi: 10.1038/s41576-019-0093-7. PMID: 30696980

[12] Marx V . Method of the Year: spatially resolved transcriptomics. Nat Methods. (2021) 18:9–14. doi: 10.1038/s41592-020-01033-y. PMID: 33408395

[13] Tripathy BB Samal KC . Protein-deficient diabetes mellitus in India. Int J Diabetes Dev Ctries. (1993) 13:1–3. doi: 10.1016/j.cels.2016.08.011

[14] Huh KB Lee HC Kim HM . Immunogenetic and nutritional profile in insulin-using youth-onset diabetics in Korea. Diabetes Res Clin Pract. (1992) 16:63–70. doi: 10.1016/0168-8227(92)90136-f. PMID: 1576933

[15] Bajaj JS Subba RG . Malnutrition-related diabetes mellitus. In: Brodoff BN Bleicher SJ , editors.World Book of Diabetes in Practice, vol. 3 (1988). p. 24–31.

[16] Kibirige D Lumu W Jones AG . Understanding the manifestation of diabetes in sub-Saharan Africa. Clin Diabetes Endocrinol. (2019) 5:2. doi: 10.1186/s40842-019-0077-8 30783538 PMC6376682

[17] Kibirige D Sekitoleko I Lumu W . Understanding the pathogenesis of lean non-autoimmune diabetes in an African population. Diabetologia. (2022) 65:675–83. doi: 10.1007/s00125-021-05644-8. PMID: 35138411 PMC8894297

[18] Bavuma C Sahabandu D Musafiri S . Atypical forms of diabetes mellitus in Africans. J Glob Health. (2019) 9:20401. doi: 10.7189/jogh.09.020401. PMID: 31673335 PMC6818125

[19] Tripathy BB . Observations on clinical patterns of diabetes mellitus in India. Diabetes. (1965) 14:404–12. doi: 10.2337/diab.14.7.404. PMID: 14318588

[20] Samal KC Kanungo A Sanjeevi CB . Clinicoepidemiological and biochemical profile of malnutrition-modulated diabetes mellitus. Ann N Y Acad Sci. (2002) 958:131–7. doi: 10.1111/j.1749-6632.2002.tb02955.x. PMID: 12021092

[21] George AM Jacob AG Fogelfeld L . Lean diabetes mellitus: an emerging entity in the era of obesity. World J Diabetes. (2015) 6:613–20. doi: 10.4239/wjd.v6.i4.613. PMID: 25987958 PMC4434081

[22] Abdulkadir J Mengesha B Gabriel ZW . Clinical and hormonal profile in malnutrition-related diabetes mellitus. Diabetologia. (1990) 33:222–7. doi: 10.1007/bf00404800. PMID: 2112100

[23] Lebovitz HE Banerji MA . Ketosis-prone diabetes (Flatbush diabetes). Curr Diabetes Rep. (2018) 18(11):120. doi: 10.1007/s11892-018-1075-4 PMC6182625 30280274

[24] Diagnosis and classification of diabetes: standards of care in diabetes—2026. Diabetes Care. (2026) 49:S27–49. doi: 10.2337/dc26-s002. PMID: 41358893 PMC12690183

[25] Okorie KC Osuagwu SC . Experiences of Adolescents with Health Promotion in the Management of Type 1 Diabetes: a literature review.

[26] Młynarska E Czarnik W Dzieża N Jędraszak W Majchrowicz G Prusinowski F . Type 2 diabetes mellitus: new pathogenetic mechanisms, treatment and the most important complications. Int J Mol Sci. (2025) 26(3):1094. doi: 10.3390/ijms26031094 39940862 PMC11817707

[27] Hawa MI Buchan AP Ola T Wun CC DeMicco DA Bao W . LADA and CARDS: a prospective study of clinical outcome in established adult-onset autoimmune diabetes. Diabetes Care. (2014) 37:1643–9. doi: 10.2337/dc13-2383. PMID: 24722498

[28] Riddle MC Philipson LH Rich SS Carlsson A Franks PW Greeley SA . Monogenic diabetes: from genetic insights to population-based precision in care. Reflections from a diabetes care editors’ expert forum. Diabetes Care. (2020) 43:3117–28. doi: 10.2337/dci20-0065. PMID: 33560999 PMC8162450

[29] Katoch OR . Determinants of malnutrition among children. Nutrition. (2022) 96:111565. doi: 10.1016/j.nut.2021.111565 35066367

[30] Rytter MJ Kolte L Briend A . The immune system in children with malnutrition. PLoS One. (2014) 9(8):e105017. doi: 10.1371/journal.pone.010501 25153531 PMC4143239

[31] Stevens GA Beal T Mbuya MNN . Micronutrient deficiencies among women and children worldwide. Lancet Glob Health. (2022) 10:e1590–9. doi: 10.1016/s2214-109x(22)00367-9. PMID: 36240826 PMC10918648

[32] Sandjaja S Budiman B Harahap H . Food consumption and nutritional status of Indonesian children. Br J Nutr. (2013) 110:S11–20. doi: 10.1017/s0007114513002109. PMID: 24016762

[33] Rojroongwasinkul N Kijboonchoo K Wimonpeerapattana W Purttiponthanee S Yamborisut U Boonpraderm A . SEANUTS: the nutritional status and dietary intakes of 0.5 12-year-old Thai children. Br J Nutr. (2013) 110(Suppl 3):S36–44. doi: 10.1017/S0007114513002110 24016765

[34] Nguyen BKL Le Thi H Nguyen Do VA Tran TN Nguyen Huu C Thanh Do T . Double burden of undernutrition and overnutrition in Vietnam in children aged 0.5 11 years: the SEANUTS study. Br J Nutr. (2013) 110(Suppl 3):S45–56. doi: 10.1017/S0007114513002080. PMID: 24016766

[35] Poh BK Ng BK Haslinda MD . Nutritional status of Malaysian children. Br J Nutr. (2013) 110:S21–35. doi: 10.1017/s0007114513002092. PMID: 24016764

[36] Mithal A Wahl DA Bonjour JP . Global vitamin D status and determinants. Osteoporos Int. (2009) 20:1807–20. doi: 10.1007/s00198-009-1030-y. PMID: 19543765

[37] Pongcharoen T Rojroongwasinkul N Tuntipopipat S . Triple burden of malnutrition among Thai children. Public Health Nutr. (2024) 27:e152. doi: 10.1017/s1368980024000053. PMID: 38250788 PMC11617422

[38] Poh BK Wong JE Lee ST . Triple burden of malnutrition in Malaysian children. Public Health Nutr. (2024) 27:e151. doi: 10.1017/s1368980023002239. PMID: 37932916 PMC11617412

[39] Govers C Calder PC Savelkoul HFJ . Nutrition and viral respiratory tract infections. Front Immunol. (2022) 13:841532. doi: 10.3389/fimmu.2022.841532. PMID: 35296080 PMC8918570

[40] Budu E Armah-Ansah EK Gyawu NO . Inequalities in child malnutrition in Ghana. BMC Public Health. (2025) 25:2954. doi: 10.1186/s12889-025-24356-x. PMID: 40866872 PMC12382195

[41] World Health Organization . Compendium of WHO and other UN guidance in health and environment. Geneva: WHO (2024).

[42] World Health Organization United Nations Children’s Fund . Joint child malnutrition estimates. Geneva: WHO (2024).

[43] Subramanian S Huq S Yatsunenko T . Persistent gut microbiota immaturity in malnourished children. Nature. (2014) 510:417–21. doi: 10.1038/nature13421. PMID: 24896187 PMC4189846

[44] Bellanti F Lo Buglio A Quiete S . Malnutrition in hospitalized older patients. Nutrients. (2022) 14:910. doi: 10.3390/jcm9061898. PMID: 35215559 PMC8880030

[45] Sharma S Tripathi P . Gut microbiome and type 2 diabetes. J Nutr Biochem. (2019) 63:101–8. doi: 10.1007/978-3-030-53370-0_21. PMID: 30366260

[46] Alshehri D Saadah O Mosli M . Gut microbiota dysbiosis in inflammatory bowel disease. Bosn J Basic Med Sci. (2021) 21:270–6. doi: 10.17305/bjbms.2020.5016. PMID: 33052081 PMC8112554

[47] Nesci A Carnuccio C Ruggieri V . Gut microbiota and cardiovascular disease. Int J Mol Sci. (2023) 24(10):9087. doi: 10.3390/ijms24109087 37240434 PMC10219307

[48] Vimal J Himal I Kannan S . Microbial dysbiosis in carcinogenesis. Indian J Med Res. (2020) 152:553–61. doi: 10.4103/ijmr.ijmr_1026_18. PMID: 34145094 PMC8224166

[49] Ahmed GK Ramadan HK Elbeh K . Gut microbiota and psychiatric disorders. Middle East Curr Psychiatry. (2024) 31:2. doi: 10.1186/s43045-024-00395-9. PMID: 38164791

[50] Kane AV Dinh DM Ward HD . Childhood malnutrition and the intestinal microbiome. Pediatr Res. (2015) 77:256–62. doi: 10.1038/pr.2014.179. PMID: 25356748 PMC4476274

[51] Stumpf F Keller B Gressies C . Inflammation and nutrition. Nutrients. (2023) 15:1159. doi: 10.1002/jpen.2534. PMID: 36904164 PMC10005147

[52] Yatsunenko T Rey FE Manary MJ . Human gut microbiome across age and geography. Nature. (2012) 486:222–7. doi: 10.1038/nature11053. PMID: 22699611 PMC3376388

[53] Human Microbiome Project Consortium . A framework for human microbiome research. Nature. (2012) 486:215–21. doi: 10.1038/nature11209. PMID: 22699610 PMC3377744

[54] Zoghi S Sadeghpour Heravi F Nikniaz Z . Gut microbiota and childhood malnutrition. Eng Life Sci. (2024) 24:2300070. doi: 10.1002/elsc.202300070 38708416 PMC11065333

[55] Yang L Chu Z Liu M . Amino acid metabolism in immune cells. J Hematol Oncol. (2023) 16:59. doi: 10.1186/s13045-023-01453-1. PMID: 37277776 PMC10240810

[56] Weichhart T Hengstschläger M Linke M . Regulation of innate immune cell function by mTOR. Nat Rev Immunol. (2015) 15:599–614. doi: 10.1038/nri3901. PMID: 26403194 PMC6095456

[57] Powell JD Pollizzi KN Heikamp EB . Regulation of immune responses by mTOR. Annu Rev Immunol. (2012) 30:39–68. doi: 10.1146/annurev-immunol-020711-075024. PMID: 22136167 PMC3616892

[58] Jiao Y Wu L Huntington ND . Crosstalk between gut microbiota and innate immunity. Front Immunol. (2020) 11:282. doi: 10.3389/fimmu.2020.00282. PMID: 32153586 PMC7047319

[59] Natividad JM Verdu EF . Modulation of intestinal barrier by microbiota. Pharmacol Res. (2013) 69(1):42–51. doi: 10.1016/j.phrs.2012.10.007 23089410

[60] Reddy VI Raghuramulu N Bhaskaram C . Secretory IgA in protein-calorie malnutrition. Arch Dis Child. (1976) 51:871–4. doi: 10.1136/adc.51.11.871. PMID: 827242 PMC1546061

[61] Chakaroun RM Massier L Kovacs P . Gut microbiome and metabolic disease. Nutrients. (2020) 12(4):1082. doi: 10.3390/nu12041082 32295104 PMC7230435

[62] Tourkochristou E Triantos C Mouzaki A . Nutritional factors and immunological outcomes. Front Immunol. (2021) 12:665968. doi: 10.3389/fimmu.2021.665968. PMID: 34135894 PMC8201077

[63] Rytter MJH Kolte L Briend A Friis H Christensen VB . The immune system in children with malnutrition. PLoS One. (2014) 9(8):e105017. doi: 10.1371/journal.pone.0105017 25153531 PMC4143239

[64] Cui H Cruz-Correa M Giardiello FM . Loss of IGF2 imprinting and colorectal cancer risk. Science. (2003) 299:1753–5. doi: 10.1016/s0016-5085(03)82783-x 12637750

[65] Fowden AL Ward JW Wooding FBP . Programming placental nutrient transport capacity. J Physiol. (2006) 572:5–15. doi: 10.1113/jphysiol.2005.104141. PMID: 16439433 PMC1779642

[66] Timp W Levchenko A Feinberg AP . Epigenetic progenitor lesions in cancer. Cell Cycle. (2009) 8:383–90. doi: 10.4161/cc.8.3.7542. PMID: 19177016 PMC6275123

[67] Ling C Del Guerra S Lupi R . Epigenetic regulation of PPARGC1A in human diabetic islets. Diabetologia. (2008) 51:615–22. doi: 10.1007/s00125-007-0916-5. PMID: 18270681 PMC2270364

[68] McGee SL Hargreaves M . Histone modifications and exercise adaptations. J Appl Physiol. (2011) 110:258–63. doi: 10.1152/japplphysiol.00979.2010. PMID: 21030677

[69] Raychaudhuri N Raychaudhuri S Thamotharan M . Histone code modifications repress GLUT4. J Biol Chem. (2008) 283:13611–26. doi: 10.1074/jbc.m800128200. PMID: 18326493 PMC2376250

[70] McGregor RA Choi MS . microRNAs in adipogenesis and obesity. Curr Mol Med. (2011) 11:304–16. doi: 10.2174/156652411795677990. PMID: 21506921 PMC3267163

[71] Poy MN Eliasson L Krutzfeldt J . Pancreatic islet-specific microRNA regulates insulin secretion. Nature. (2004) 432(7014):226–30. doi: 10.1038/nature03076. PMID: 15538371

[72] Zhang X Wang X Yuan Z Radford SJ Liu C Libutti SK . Amino acids-Rab1A-mTORC1 signaling controls whole-body glucose homeostasis. Cell Rep. (2021) 34(11). doi: 10.1016/j.celrep.2021.108830. PMID: 33730578 PMC8062038

[73] Fan J Yuan Z Burley SK Libutti SK Zheng XS . Amino acids control blood glucose levels through mTOR signaling. Eur J Cell Biol. (2022) 101:151240. doi: 10.1016/j.ejcb.2022.151240. PMID: 35623230 PMC10035058

[74] Fan J Zhang X Zhang J Zhao T Burley SK Zheng XS . PDX1 phosphorylation at S61 by mTORC1 links nutrient signaling to β cell function and metabolic disease. Cell Rep. (2026) 45(1). doi: 10.1016/j.celrep.2025.115634. PMID: 41528843 PMC12949489

[75] Zhao T Fan J Abu-Zaid A Burley SK Zheng XS . Nuclear mTOR signaling orchestrates transcriptional programs underlying cellular growth and metabolism. Cells. (2024) 13:781. doi: 10.3390/cells13090781. PMID: 38727317 PMC11083943

[76] Vaiserman AM . Early-life nutritional programming of type 2 diabetes. Nutrients. (2017) 9:236. doi: 10.3390/nu9030236. PMID: 28273874 PMC5372899

[77] McDonagh M Ali L Kahn A . Oxidative stress in malnutrition-related diabetes. Biochem Soc Trans. (1997) 25(1):146S. doi: 10.1042/bst025146s 9057044

[78] Francis-Emmanuel PM Thompson DS Barnett AT . Glucose metabolism in adult survivors of severe acute malnutrition. J Clin Endocrinol Metab. (2014) 99:2233–40. doi: 10.1210/jc.2013-3511. PMID: 24517147

[79] Babu S Mishra PR Ravi PK . Histomorphogenesis of pancreatic islets amidst maternal anaemia. Anat Cell Biol. (2025). doi: 10.5115/acb.24.187. PMID: 40263102 PMC12519009

[80] Brooks SE Golden MHN Payne-Robinson HM . Ultrastructure of islets in protein-energy malnutrition. West Indian Med J. (1993) 42:101–5. 8273316

[81] Barker DJP Hales CN Fall CHD Osmond C Phipps K Clark PMS . Type 2 (non-insulin-dependent) diabetes mellitus, hypertension and hyperlipidaemia (syndrome X): relation to reduced fetal growth. Diabetologia. (1993) 36(1):62–67. doi: 10.1007/BF00399095 8436255

[82] Prajitno JH Sutanto H . Type 5 diabetes as a malnutrition-driven crisis. J Diabetes Metab Disord. (2025) 24(2):162. doi: 10.1007/s40200-025-01620-4 40657327 PMC12246272

[83] Mertz W . Trace elements and minerals in diabetes. In: Brodoff BN Bleicher SJ , editors.Diabetes Mellitus and Obesity. Williams & Wilkins, Baltimore (1982). p. 343–8.

[84] Benjamin JS Culpepper CB Brown LD . Chronic anemic hypoxemia attenuates insulin secretion. Am J Physiol Regul Integr Comp Physiol. (2017) 312:R492–500. doi: 10.1152/ajpregu.00484.2016. PMID: 28100476 PMC5407078

[85] Sanjeevi CB Kanungo A Samal KC . Immunogenetic studies on malnutrition-modulated diabetes mellitus. Ann N Y Acad Sci. (2002) 958:144–7. doi: 10.1111/j.1749-6632.2002.tb02957.x. PMID: 12021094

[86] Chiang YT Jin T . p21-activated protein kinases in glucose homeostasis. Am J Physiol Endocrinol Metab. (2014) 306:E707–22. doi: 10.1152/ajpendo.00506.2013. PMID: 24368667

[87] Jebasingh F Thomas N . Barker hypothesis and hypertension. Front Public Health. (2022) 9:767545. doi: 10.3389/fpubh.2021.767545. PMID: 35127619 PMC8814110

[88] Liepert M Alhaqqan S Husain A . Anti-glutamic acid decarboxylase antibodies in autoimmune diabetes diagnosis. Clin Biochem. (2025) 135:11. doi: 10.1016/j.clinbiochem.2024.110842. PMID: 39551460

[89] Lee H Park J Kim YH . Integrated spatial transcriptomics reveals β-cell heterogeneity and stress pathway activation in human diabetic pancreas. Nat Metab. (2022) 4(9):1235–48. doi: 10.1038/s42255-022-00637-4

[90] Camunas-Soler J Dai XQ Hang Y . Patch-seq links single-cell transcriptomes to human islet dysfunction in diabetes. Cell Metab. (2020) 31(5):1017–1031.e4. doi: 10.1016/j.cmet.2020.04.005. PMID: 32302527 PMC7398125

[91] Moses L Pachter L . Museum of spatial transcriptomics. Nat Methods. (2022) 19:534–46. doi: 10.1038/s41592-022-01409-2. PMID: 35273392

[92] Duan H Cheng T Cheng H . Spatially resolved transcriptomics: advances and applications. Blood Sci. (2023) 5:1–4. doi: 10.1097/bs9.0000000000000141. PMID: 36742187 PMC9891446

[93] Lee JH Daugharthy ER Scheiman J Kalhor R Yang JL Ferrante TC . Highly multiplexed subcellular RNA sequencing in situ. Science. (2014) 343:1360–3. doi: 10.1126/science.1250212. PMID: 24578530 PMC4140943

[94] Junker JP Noel ES Guryev V Peterson KA Shah G Huisken J . Genome-wide RNA tomography in the zebrafish embryo. Cell. (2014) 159:662–75. doi: 10.1016/j.cell.2014.09.038. PMID: 25417113

[95] Peng G Suo S Chen J Chen W Liu C Yu F . Spatial transcriptome for the molecular annotation of lineage fates and cell identity in mid-gastrula mouse embryo. Dev Cell. (2016) 36:681–97. doi: 10.1016/j.devcel.2020.11.018. PMID: 27003939

[96] Vandereyken K Sifrim A Thienpont B Voet T . Methods and applications for single-cell and spatial multi-omics. Nat Rev Genet. (2023) 24:494–515. doi: 10.1038/s41576-023-00580-2. PMID: 36864178 PMC9979144

[97] Zhao T Chiang ZD Morriss JW LaFave LM Murray EM Del Priore I . Spatial genomics enables multi-modal study of clonal heterogeneity in tissues. Nature. (2022) 601:85–91. doi: 10.1038/s41586-021-04217-4. PMID: 34912115 PMC9301586

[98] Deng Y Bartosovic M Ma S Zhang D Kukanja P Xiao Y . Spatial profiling of chromatin accessibility in mouse and human tissues. Nature. (2022) 609:375–83. doi: 10.1038/s41586-022-05094-1. PMID: 35978191 PMC9452302

[99] Saka SK Wang Y Kishi JY Zhu A Zeng Y Xie W . Immuno-SABER enables highly multiplexed and amplified protein imaging in tissues. Nat Biotechnol. (2019) 37:1080–90. doi: 10.1038/s41587-019-0207-y. PMID: 31427819 PMC6728175

[100] Hu T Allam M Cai S Henderson W Yueh B Garipcan A . Single-cell spatial metabolomics with cell-type specific protein profiling for tissue systems biology. Nat Commun. (2023) 14:8260. doi: 10.1038/s41467-023-43917-5. PMID: 38086839 PMC10716522

[101] Unsihuay D Mesa Sanchez D Laskin J . Quantitative mass spectrometry imaging of biological systems. Annu Rev Phys Chem. (2021) 72:307–29. doi: 10.1146/annurev-physchem-061020-053416. PMID: 33441032 PMC8161172

[102] Jiang F Zhou X Qian Y Zhu M Wang L Li Z . Simultaneous profiling of spatial gene expression and chromatin accessibility for mouse brain development. bioRxiv (2022). p. 2022–03. doi: 10.1101/2022.03.23.485517 37231265

[103] Wagner DE Klein AM . Lineage tracing meets single-cell omics: opportunities and challenges. Nat Rev Genet. (2020) 21:410–27. doi: 10.1038/s41576-020-0223-2. PMID: 32235876 PMC7307462

[104] Erhard F Saliba AE Lusser A Toussaint C Hennig T Prusty BK . Time-resolved single-cell RNA-seq using metabolic RNA labelling. Nat Rev Methods Primers. (2022) 2:77. doi: 10.1038/s43586-022-00157-z. PMID: 37880705

[105] Fang S Chen B Zhang Y Sun H Liu L Liu S . Computational approaches and challenges in spatial transcriptomics. Genomics Proteomics Bioinf. (2023) 21:24–47. doi: 10.1016/j.gpb.2022.10.001. PMID: 36252814 PMC10372921

[106] Satija R Farrell JA Gennert D Schier AF Regev A . Spatial reconstruction of single-cell gene expression data. Nat Biotechnol. (2015) 33:495–502. doi: 10.1038/nbt.3192. PMID: 25867923 PMC4430369

[107] Stringer C Wang T Michaelos M Pachitariu M . Cellpose: a generalist algorithm for cellular segmentation. Nat Methods. (2021) 18:100–6. doi: 10.1038/s41592-020-01018-x. PMID: 33318659

[108] Townes FW Engelhardt BE . Nonnegative spatial factorization applied to spatial genomics. Nat Methods. (2023) 20:229–38. doi: 10.1038/s41592-022-01687-w. PMID: 36587187 PMC9911348

[109] Kiemen AL Braxton AM Grahn MP Han KS Babu JM Reichel R . CODA: quantitative 3D reconstruction of large tissues at cellular resolution. Nat Methods. (2022) 19:1490–9. doi: 10.1038/s41592-022-01650-9. PMID: 36280719 PMC10500590

[110] Richardson L Venkataraman S Stevenson P Yang Y Moss J Graham L . EMAGE mouse embryo spatial gene expression database: 2014 update. Nucleic Acids Res. (2014) 42:D835–44. doi: 10.1093/nar/gkt1155. PMID: 24265223 PMC3965061

[111] Karimi K Fortriede JD Lotay VS Burns KA Wang DZ Fisher ME . Xenbase: a genomic, epigenomic and transcriptomic model organism database. Nucleic Acids Res. (2018) 46:D861–8. doi: 10.1093/nar/gkx936. PMID: 29059324 PMC5753396

[112] Regev A Teichmann SA Lander ES Amit I Benoist C Birney E . The human cell atlas. elife. (2017) 6:e27041. doi: 10.1038/s41580-020-0267-3. PMID: 29206104 PMC5762154

[113] Snyder MP Lin S Posgai A Atkinson M Regev A Rood J . Mapping the human body at cellular resolution--the NIH Common Fund Human BioMolecular Atlas program. arXiv preprint arXiv:1903.07231. (2019). 10.1038/s41586-019-1629-x PMC6800388 31597973

[114] Xu Z Wang W Yang T Li L Ma X Chen J . STOmicsDB: a comprehensive database for spatial transcriptomics data sharing, analysis and visualization. Nucleic Acids Res. (2024) 52:D1053–61. doi: 10.1093/nar/gkad933. PMID: 37953328 PMC10767841

[115] Fan Z Chen R Chen X . SpatialDB: a database for spatially resolved transcriptomes. Nucleic Acids Res. (2020) 48:D233–7. doi: 10.1093/nar/gkz934. PMID: 31713629 PMC7145543

[116] Li Y Dennis S Hutch MR Ding Y Zhou Y Pillai M . SOAR elucidates disease mechanisms and empowers drug discovery through spatial transcriptomics. Nat Biotechnol. (2024). doi: 10.1038/s41587-024-02143-2 PMC13109963 40498826

[117] Yuan Z Pan W Zhao X Zhao F Xu Z Li X . SODB facilitates comprehensive exploration of spatial omics data. Nat Methods. (2023) 20:387–99. doi: 10.1038/s41592-023-01773-7. PMID: 36797409

[118] Rozenblatt-Rosen O Regev A Oberdoerffer P Nawy T Hupalowska A Rood JE . The human tumor atlas network: charting tumor transitions across space and time at single-cell resolution. Cell. (2020) 181:236–44. doi: 10.1016/j.cell.2020.03.053. PMID: 32302568 PMC7376497

[119] Sunkin SM Ng L Lau C Dolbeare T Gilbert TL Thompson CL . Allen Brain Atlas: an integrated spatio-temporal portal for exploring the central nervous system. Nucleic Acids Res. (2012) 41:D996–1008. doi: 10.1093/nar/gks1042. PMID: 23193282 PMC3531093

[120] Zhang J Yin J Heng Y Xie K Chen A Amit I . Spatiotemporal omics-refining the landscape of precision medicine. Life Med. (2022) 1:84–102. doi: 10.1093/lifemedi/lnac053. PMID: 39871933 PMC11749813

[121] Ritari J Salojärvi J Lahti L de Vos WM . Improved taxonomic assignment of human intestinal 16S rRNA sequences by a dedicated reference database. BMC Genomics. (2015) 16:1056. doi: 10.1186/s12864-015-2265-y. PMID: 26651617 PMC4676846

[122] Wright ES Yilmaz LS Corcoran AM Ökten HE Noguera DR . Automated design of probes for rRNA-targeted fluorescence in situ hybridization reveals the advantages of using dual probes for accurate identification. Appl Environ Microbiol. (2014) 80:5124–33. doi: 10.1128/aem.01685-14. PMID: 24928876 PMC4135741

[123] Zhu B Bai Y Yeo YY Lu X Rovira-Clavé X Chen H . A multi-omics spatial framework for host-microbiome dissection within the intestinal tissue microenvironment. Nat Commun. (2025) 16. doi: 10.1038/s41467-025-56184-7. PMID: 39890778 PMC11785740

[124] Melton R Jimenez S Elison W Tucciarone L Howell A Wang G . Single-cell multiome and spatial profiling reveals pancreas cell type–specific gene regulatory programs of type 1 diabetes progression. Sci Adv. (2025) 11:eady0080. doi: 10.1126/sciadv.ady0080. PMID: 40929272 PMC12422192

[125] Howell N Weiss Z Bonnycastle LL Grenko CM Randazzo D Dampier CH . A spatial transcriptomics dataset of pancreas sections in normal glucose tolerance and type 2 diabetic donors. Sci Data. (2025) 12:1526. doi: 10.1038/s41597-025-05450-6. PMID: 40890166 PMC12402493

[126] Qian Y Xiong S Li L Sun Z Zhang L Yuan W . Spatial multiomics atlas reveals smooth muscle phenotypic transformation and metabolic reprogramming in diabetic macroangiopathy. Cardiovasc Diabetol. (2024) 23:358. doi: 10.1186/s12933-024-02458-x. PMID: 39395983 PMC11471023

[127] Mou L Wang TB Chen Y Luo Z Wang X Pu Z . Single-cell genomics and spatial transcriptomics in islet transplantation for diabetes treatment: advancing towards personalized therapies. Front Immunol. (2025) 16:1554876. doi: 10.3389/fimmu.2025.1554876. PMID: 40051625 PMC11882877

